# Auricular therapy as adjunctive treatment for pediatric attention deficit hyperactivity disorder: a scoping review

**DOI:** 10.3389/fped.2026.1829733

**Published:** 2026-06-26

**Authors:** Siqi Wang, Yongfen Jin, Lei Hong, Qijun You, Haixia Zhang

**Affiliations:** 1 Pediatrics Department, Huzhou Nanxun District Traditional Chinese Medicine Hospital, Huzhou, China; 2 Pediatrics Department, Jincheng Subdistrict Community Health Service Center, Hangzhou, China

**Keywords:** attention deficit hyperactivity disorder, auricular therapy, meta-analysis, pediatric patients, scoping review

## Abstract

**Objective:**

Up to now, attention deficit hyperactivity disorder (ADHD), a prevalent neurodevelopmental disorder in children, remains controversial efficiency of auricular therapy as an adjunctive treatment. Our study aims to synthesize the current evidence from RCTs and NRCTs by conducting qualitative description and quantitative synthesis, in order to draw a further conclusion for ADHD clinical practice.

**Methods:**

A total of 7 databases were searched from inception to July 15, 2025. Afterwards, both randomized and non-randomized controlled trials were included, where clinical outcomes were not restricted. After screening eligible studies, risk of bias was assessed using the Cochrane ROB2 and ROBINS-I tools respectively. Then, the characteristics of included studies were tabulated and meta-analyses was conducted.

**Results:**

A total of 21 studies involving 2,270 participants were included. From our main findings, adjunctive auricular therapy could significantly improve scores of Conners' Parent Rating Scale, Hamilton Anxiety Scale and Hamilton Depression Rating Scale (all *P* < 0.05), and enhance quality of life questionnaire score (*P* < 0.05). And a non-significant trend toward higher overall efficacy rate was observed (*P* > 0.05), while no serious adverse events were reported. Whereas, the risk of bias in included studies was assessed as low-and-moderate-certainty evidence.

**Conclusion:**

This work indicates promising effects of auricular therapy in alleviating ADHD-related symptoms among children with long-term interventions, but current evidence is still insufficient to support a 100% positive conclusion due to several limitations. And future studies with rigorously designed pragmatic randomized controlled trials should be encouraged, under real world settings.

## 1 Introduction

Attention deficit hyperactivity disorder (ADHD) is a neurodevelopmental disorder with onset during childhood, which was characterized by age-inappropriate levels of hyperactivity, impulsivity, and inattention (1, 2). ADHD frequently co-occurs with conditions (e.g., obsessive-compulsive disorder, tic disorders) and significantly impacts kids' learning ability, family and social relationships, and psychological functioning (3, 4). According to recent research, the global and domestic prevalence of ADHD were 5.29% (5) and 5.6%–6.26% (1, 6) respectively. And the incidence of ADHD in school-age and adolescents were 4.2%–6.4% and 3.1%–11.1% respectively (5). Moreover, approximately 40%–70% of adolescents with ADHD continue to experience impairing symptoms until their adulthood (3, 7, 8), which is a typically lifelong illness. Hence, early intervention in ADHD is crucial (9).

Regarding the treatment of ADHD, non-pharmacological interventions are the preferable choice for children below the age of 5–6 years (2, 10), whose secondary therapy was combination of pharmacological and non-pharmacological treatments (2, 10, 11). Central nervous system (CNS) stimulants (e.g., methylphenidate, dextroamphetamine) are the first-line medications for ADHD, while non-CNS stimulants (e.g., atomoxetine hydrochloride and clonidine) are recommended as second-line drugs (11, 12). However, CNS stimulants may cause side effects including cardiovascular events and insomnia, whereas non-CNS stimulants may result in gastrointestinal adverse events such as nausea, vomiting, decreased appetite, and abdominal pain, along with other adverse reactions including drowsiness, fatigue, irritability, headache, hypotension, and bradycardia (13). Additionally, complementary and alternative medicine approaches also play an important role in ADHD treatment, including taking Chinese medicines (CMs), Tuina, acupuncture, auricular therapy (9). Among them, auricular therapy features distinct advantages including simplicity, safety and minimal discomfort, making it highly acceptable to patients, especially children. It works by stimulating specific auricular acupoints to treat and prevent diseases. Furthermore, its theoretical basis is mainly derived from the meridian theory and biological holographic theory of traditional Chinese medicine (TCM). Up to now, a variety of studies have reported that auricular therapy as an adjunctive treatment can improve sleep quality, reduce behavioral problems, and promote quality of life, behavior as well as negative emotions, without increasing adverse reactions among ADHD kids (14–22). Whereas, there were some researchers who insisted on the opposite conclusion that the incorporation of auricular therapy does not guarantee improved clinical outcomes (23–26).

While a certain number of researches have explored auricular therapy for pediatric ADHD, which indicates either effective or ineffective improvements of ADHD-related symptoms, further evidence synthesis ought to be generated via a scoping review to answer this question. Against this backdrop, our study aims to investigate auricular therapy as an adjunctive treatment for improving pediatric ADHD and to facilitate its integration into clinical practice.

## 2 Methods

### 2.1 Systematic retrieval and search strategies

A systematic retrieval of published researches from common databases [viz., PubMed, Embase, Web of science, EBSCO, VIP Database for Chinese Technical Periodicals (VIP), China National Knowledge Infrastructure (CNKI), WanFang Database] from their inception to July 15, 2025, was conducted. In accordance with the PRISMA-ScR guidelines (27), a scoping review does not require a complete PICO framework. Accordingly, our study only limited P (Population) and I (Intervention). And our search strategy consisted of 2 components: (1) Targeted disease (ADHD); (2) Intervention (auricular therapy), whose additional search terms and detailed strategies were described in Supplementary Table S1. Moreover, unindexed journals as well as clinical trial registries were not searched.

### 2.2 Inclusion and exclusion criteria of studies

First at all, studies focused on patients who were under 18 years of age (28) and diagnosed as ADHD, Attention Deficit Disorders with Hyperactivity, Hyperkinetic Syndrome, Attention Deficit Disorder, or Minimal Brain Dysfunction were included (29). Subsequently, the excluded studies were as followed: (1) Non-English or non-Chinese studies; (2) Non-clinical studies (e.g., *in-vitro* or/and *in-vivo* experiments); (3) Clinical studies lacking control/comparison group (e.g., case reports, case series); (4) Studies involving participants with other comorbidities (e.g., tic disorders) that may interfere comparison; (5) Studies with missing or incomplete data for outcome; (6) Studies with unbalanced characteristics at baseline; (7) Studies utilizing non-validated, self -defined criteria for efficacy assessment; (8) Studies involved with auricular therapy in both experimental group and control group.

### 2.3 Study selection and data extraction

All records identified through systematic search were retrieved and imported into EndNote 20. After duplications were removed, all identified articles were accessed for relevance via their titles and abstracts independently by three reviewers (SQ Wang, L Hong, HX Zhang). Then, full-text screening for eligibility was executed according to pre-set included and excluded criteria by aforesaid reviewers. Afterwards, the core data were extracted from the included studies, using Excel table aligned with research objective including sample size, number of experimental or control group, interventions, etc. Any disagreements regarding aforementioned process would be resolved through a discussion with a third reviewer (YF Jin). In the case of missing data, the authors were contacted via email (if applicable).

### 2.4 Data synthesis and appraisal of methodological quality

As to the same comparisons among included studies, we conducted meta-analyses using a random effect model in view of highly individualized TCM treatment, especially for some contradictory results from different studies. And the methodological quality of randomized controlled trials (RCTs) and non-randomized control trials (NRCTs) were respectively assessed using the Cochrane Risk of Bias Tool—ROB2 and ROBINS-I (Risk of bias in non-randomized studies of interventions). On one hand, the risk of bias graph and traffic light plot were visualized using RevMan 5.4; On the other, ROBINS-I was used for evaluating the methodological quality of non-randomized control trials via EXCEL.

### 2.5 Reporting and registration

In accordance with the PRISMA-ScR statement (27), the complete reporting checklist for this scoping review is accessible as Supplementary Table S2. Since the registered protocol for scoping review was not available to international prospective register of systematic review (PROSPERO), our study could not obtain a registration number.

## 3 Results

### 3.1 Search results

**Figure 1:**
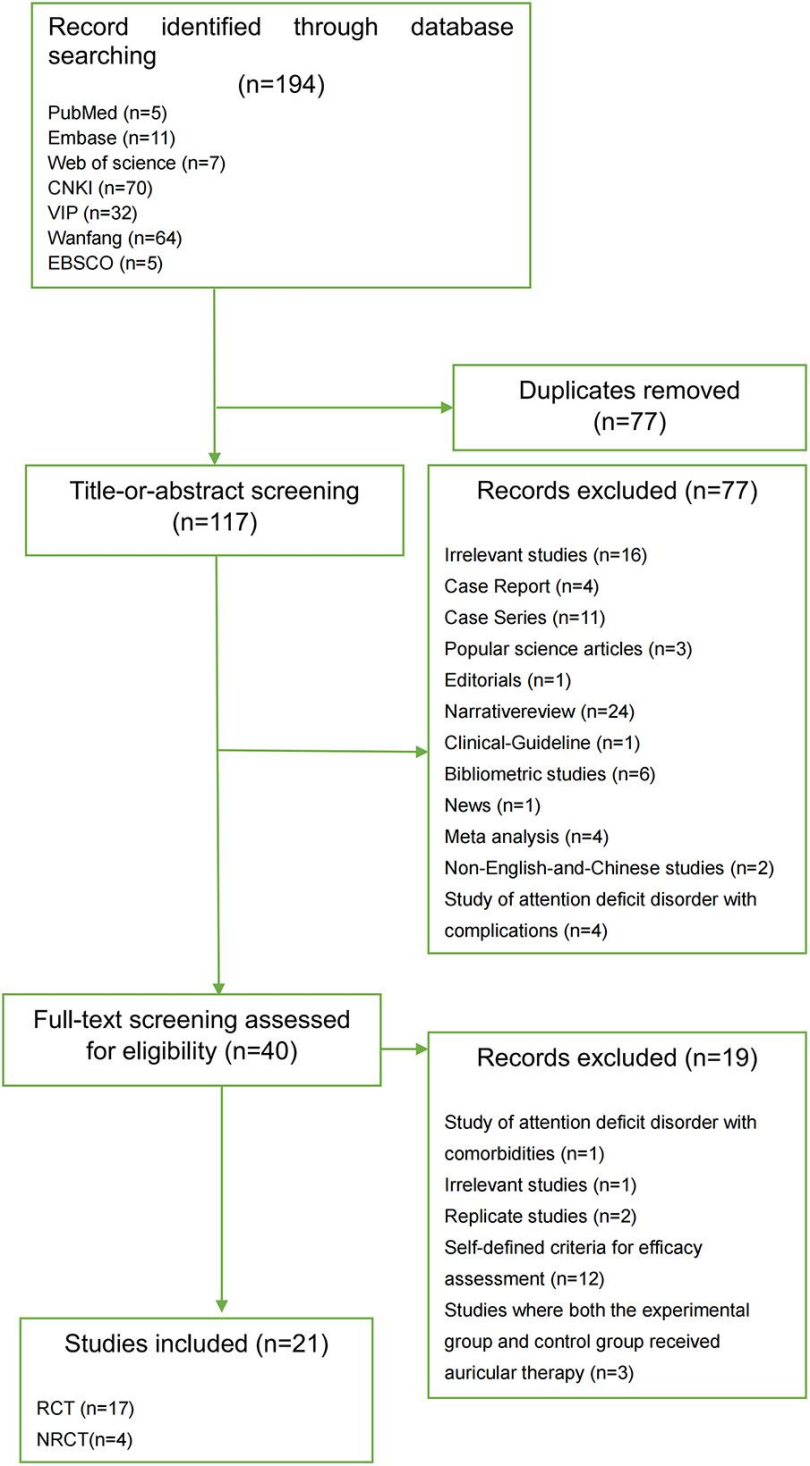
Flow diagram representing process of identifying eligible studies.

Our search returned 194 records from abovementioned databases. And we identified 117 potentially relevant studies for screening after excluding duplicates. A total of 77 studies were excluded subsequent to title-and-abstract screening, while remaining 40 studies were assessed during full-text screening. Eventually, a total of 21 met the inclusion and exclusion criteria (Figure 1), which comprised of 17 randomized controlled trials (RCTs) and 4 non-randomized control trials (NRCTs).

### 3.2 Characteristics of included studies

Above all, we summarized the characteristics of 21 included studies (14–26, 30–37) among which 16 authors' conclusion (14–16, 18–22, 30–37) towards the role of auricular therapy in adjuvant treatment of ADHD were positive, while remaining 5 studies held the opposite viewpoints (absence of statistical significance), which were reported in section 3.4.

For one thing, the included 17 RCTs involving 1,098 participants (559 in Treatment group and 539 in control group) were published between 2006 and 2025, with a female-to-male ratio of 1:2.19. And these studies were primarily conducted in China (*n* = 14) and Iran (*n* = 3); For another, the included 4 NRCTs involving 1172 participants (779 in treatment group and 393 in control group) were published between 2003 and 2024, and a female-to-male ratio of 1:3.82. And these studies were all conducted in China. Subsequently, we supplemented some missing data using statistical methods because no reply was received after contacting the authors. And the characteristics of included studies were displayed in Table 1.

**Table 1:** Characteristics of 17 included rCTs.

Study ID	Sample size(M/F)	Age	Intervention		Treatment duration	Comparison	Outcome
		(*μ* ± SD)	T	C			
Laleh Mohammadi 2025	45^a^	T:7.87 ± 1.42	Ear acupressure with seeds	Ear adhesives without seeds and pressure	4 weeks	Auricular therapy vs. Placebo	①
	(T:15/8; C:16/6)	C:9.09 ± 1.26					
Fatemeh Mahdavi 2024	60^b^	T:8.49 ± 2.11	Ear acupressure with seeds	massage at neutral points	4 weeks		②
	(T:23/7; C:23/7)	C:8.62 ± 1.61					
Marzieh Binesh 2020	44^c^	T:9.80 ± 2.00	Electroacupuncture + Ear acupressure with seeds	sham procedure	6 weeks		③④⑪
	(T:19/4; C:17/4)^d^	C:9.80 ± 2.00					
Hongrui ZHANG 2024	58^e^	T:10.57 ± 2.69	Supplementary formula of Huan-Gan-Li-Pi Granules + Ear acupressure with seeds + Other non-drug therapies^#^	Supplementary formula of Huan-Gan-Li-Pi Granules + Other non-drug therapies^#^	6 weeks	CMs + Auricular therapy vs. CMs	⑤⑥⑦
	(T:16/12; C:22/8)	C:10.10 ± 2.47					
Hong HUANG 2025	30	T:8.11 ± 1.25	Customized CM-preparations + Ear acupressure with seeds	Customized CM-preparations	3 months		③⑥⑧⑨⑩
	(T:10/5; C:9/6)	C:8.56 ± 1.32					
Yanhui ZHANG 2023	80	T:9.52 ± 1.05	Customized CM-preparations + Ear acupressure with seeds	Customized CM-preparations	3 months		③⑥⑧⑨⑩⑪
	(T:27/13; C:26/14)	C:9.46 ± 1.14					
Baoli ZHANG 2016	53	T:7.80 ± 1.50	Customized CM-preparations + Ear acupressure with seeds	Customized CM-preparations	3 months		⑥
	(T:15/11; C:14/13)	C:7.40 ± 1.30					
Yijia HU 2014	80	T:8.20 ± 1.10	Acupuncture + Ear acupressure with seeds	Methylphenidate hydrochloride	3 months	Acupuncture + Auricular therapy vs. Synthetic drugs	⑥
	(T:22/18; C:23/17)	C:8.00 ± 1.20					
Jiande LIU 2009	108	T:9.80 ± 1.70	Acupuncture + Ear acupressure with seeds	Methylphenidate hydrochloride	3 months		⑤⑥⑪
	(T:51/12; C:38/7)	C:10.60 ± 2.40					
Yuanzhao CHENG 2009	64	T:8.40 ± 1.50^f^	Acupuncture + Ear acupressure with seeds	Methylphenidate hydrochloride	3 months		⑥⑫
	(T:27/5; C:28/4)	C:8.60 ± 1.50^f^					
Yu ZHANG 2019	58	T:8.40 ± 0.99	Ear acupressure with seeds + Ningxin Granules	Methylphenidate hydrochloride	8 weeks	CMs + Auricular therapy vs. Synthetic drugs	⑤⑥⑪⑬
	(T:18/11; C:17/12)	C:8.56 ± 1.40					
Yan XU 2017	60	T:8.70 ± 1.20	Customized CM-preparations + Auricular Acupuncture	Methylphenidate hydrochloride	8 weeks		⑥⑪
	(T:19/11; C:21/9)	C:8.50 ± 0.90					
Siyi CHENG 2024	80	T:10.32 ± 1.17	Qi-Ju-Di-Huang pills/ZHi-Bai-Di-Huang pills + Methylphenidate hydrochloride/ Atomoxetine Hydrochloride + Acupuncture + Acupoint Herbal Patches + Ear acupressure with seeds + Other non-drug therapies^#^	Qi-Ju-Di-Huang pills/ZHi-Bai-Di-Huang pills + Methylphenidate hydrochloride/ Atomoxetine Hydrochloride	2-4 weeks	CMs + Synthetic drugs + Acupuncture + Auricular therapy + Acupoint Herbal Patches + Other non-drug therapies^#^ vs. CMs + Synthetic drugs	②⑤⑭
	(T:22/18; C:24/16)	C:9.65 ± 1.04					
Zhen ZHONG 2023	90	T:9.83 ± 2.10	Atomoxetine Hydrochloride + Ear Copper Bianstone Scraping + Ear acupressure with seeds	Atomoxetine Hydrochloride	2 months	Synthetic drugs + Ear scraping + Auricular therapy vs. Synthetic drugs	③⑤⑥⑦⑮⑯⑰
	(T:35/10; C:37/8)	C:9.67 ± 2.08					
Xizhi WU 2016	85	T:10.33 ± 4.04^g^	Acupuncture + Ear acupressure with seeds + Other non-drug therapies^#^	Customized CM-preparations + Other non-drug therapies^#^	3 weeks	Acupuncture + Auricular therapy vs. CMs	⑥
	(T:26/17; C:24/18)	C:10.23 ± 3.70^g^					
Shanwei HE 2013	63	T:9.64 ± 1.02	Supplementary formula of Liu-Wei-Di-Huang and Gan-Mai-Da-Zao Decoction + Acupuncture + Ear acupressure with seeds + Other non-drug therapies^#^	Supplementary formula of Liu-Wei-Di-Huang and Gan-Mai-Da-Zao Decoction + Methylphenidate hydrochloride + Other non-drug therapies^#^	2 months	CMs + Auricular therapy + Acupuncture vs. CMs + Synthetic drugs	②⑤⑥⑭
	(T:19/13; C:16/15)	C:10.45 ± 0.93					
Yue ZHUO 2006	40	T:8.00 ± 1.33^f^	Tuina massage + Ear acupressure with seeds	Methylphenidate hydrochloride	1 month	Tuina + Auricular therapy vs. Synthetic drugs	⑥⑫
	(T:17/3; C:18/2)	C:8.60 ± 1.50^f^					

M, male; F, female; *μ*, mean; SD, standard deviation; T, Treatment group; C, controlled group; CM, Chinese medicine; #, Other non-drug therapies includes psychotherapy and behavior therapy; a, a total of 52 participants were enrolled, of whom 45 completed the trial and 7 withdrew; b, a total of 70 participants were enrolled, of whom 60 completed the trial and 10 withdrew; c, a total of 50 participants were enrolled, of whom 44 completed the trial and 6 withdrew; d, According to original context, there were 23 and 21 participants remained in auricular therapy group and sham group respectively by the completion, with 36 (81.8%) males from 44 participants. Thus, we could estimate that 19 male (36 × 23/44≈19) and 4 female (23-19 = 4) participants in experiment group, while 17 male (36 × 21/44≈17) and 4 female participants (21-17 = 4) in control group; e, A total of 60 participants were enrolled, of whom 58 completed the trial and 2 withdrew; f, Since SD was not mentioned in this article, we estimated crude $SD\;=\;\frac{\max-\min}{6}$  with the an assumption that the original data followed a normal distribution where median equals the mean; g, We estimated crude mean and standard deviation of age based on the assumption that each age group were uniformly distributed around its midpoint, where $\mathrm{midpoint}\;=\;\frac{\max-\min}{2}$, $\mu \;=\;\frac{\sum_{n\;=\;i}\left(n\times \mathrm{midpoint}\right)}{n}$, and $SD\;=\;\frac{{\sum_{n\;=\;i}n\left(midpoint-\mu \right)}^{2}}{n-1}$. Since the patients were grouped into 4 sections (viz., children aged 5–7 years, 8–10 years, 11–14 years, 15–18 years), and accordingly their midpoint respectively equaled to 6, 9,12.5, 16.5. Then, the number of aforesaid age group respectively were 15,9,10,9 in treatment group and 12,13,10,7 in control group according to the study. Thus, two groups' crude *μ* and SD were respectively calculated, treatment groups *μ* = $\frac{15\times 6+9\times 9+10\times 12.5+9\times 16.5}{43}\approx 10.33$, treatment group SD = $\surd \frac{{\left(6-10.33\right)}^{2}\times 15+{\left(9-10.33\right)}^{2}\times 9+{\left(12.5-10.33\right)}^{2}\times 10+{\left(16.5-10.33\right)}^{2}\times 9}{42}\approx 4.04$, control group *μ* = $\frac{12\times 6+13\times 9+10\times 12.5+7\times 16.5}{42}\approx 10.22$, treatment group SD = $\surd \frac{{\left(6-10.22\right)}^{2}\times 12+{\left(9-10.22\right)}^{2}\times 13+{\left(12.5-10.22\right)}^{2}\times 10+{\left(16.5-10.22\right)}^{2}\times 7}{41}\approx 3.70$. ①Sleep quality score; ②Child Behavior Checklist (CBCL) score**;** ③Conners Parent Rating Scale (CPRS) score; ④Children's Somatization Inventory version Ⅳ (CSI-4) score; ⑤Conners Index of Hyperactivity (CIH) score; ⑥Overall efficacy rate; ⑦Swanson, Nolan, and Pelham Rating Scale, Version Ⅳ (SNAP-Ⅳ); ⑧Hamilton Anxiety Scale(HAMA) score; ⑨Hamilton Depression Scale(HAMD) score; ⑩Quality of Life Questionnaire (QOL Questionnaire) score; ⑪Incidence of adverse drug reaction (ADR); ⑫Relapse rate; ⑬Digit Cancellation Test score, DCT; ⑭Raven's Standard Progressive Matrices (SPM); ⑮Comprehensive attention quotient (CAQ); ⑯Wechsler Intelligence Scale for Children(WIC) score; ⑰Comprehensive response control quotient (CRCQ).

**Table 2:** Characteristics of 4 included NRCTs.

Study ID	Sample size(M/F)	Age (μ ± SD)	Intervention		Treatment duration	Comparison	Outcome
			T	C			
Yiping CUI 2024	425^a^	T:9.00 ± 0.50^b^	Tiaoxie Yizhi decoction + Ear acupressure with seeds	Atomoxetine Hydrochloride	3 months	CM + Auricular therapy vs. Synthetic drugs	①②③
	(T:254/57; C:102/12)	C:9.00 ± 0.66^b^					
Wenli WANG 2003	97	T:7.50 ± 1.58^b^	Customized CM-preparations + Ear acupressure with seeds + Other non-drug therapies^#^	Methylphenidate hydrochloride + Other non-drug therapies^#^	2 months		①
	(T:41/9; C:39/8)	C:7.00 ± 1.42^b^					
Meiying WANG 2009	58	T:9.60 ± 1.17^b^	Acupuncture + Ear acupressure with seeds + Other non-drug therapies^#^	Methylphenidate hydrochloride	1 month	Acupuncture + Auricular therapy + Other non-drug therapies^#^ vs. Synthetic drugs	①
	(T:28/10; C:16/4)	C:10.10 ± 1.33^b^					
Li Hong 2005	592	T:9.00 ± 1.67^b^	Acupuncture (Plum Blossom Needle) + Ear acupressure with seeds	Supplementary formula of Qi-Ju-Di-Huang Decoction and Supplementary formula of Yi-Guan-Jian Decoction	3 months	Acupuncture (Plum Blossom Needle) + Auricular therapy vs. CMs	①④
	(T:288/92; C:161/51)	C:8.00 ± 1.67^b^					

Footnote: M, male; F, female; μ, mean; SD, standard deviation; T, Treatment group; C, controlled group; CM, Chinese medicine; Other non-drug therapies: Psychotherapy and Behavior Therapy; a, A total of 563 participants were enrolled, of whom 425 completed the trial and 138 withdrew; b, Since SD was not mentioned in this article, we estimated $crudeSD\;=\;\frac{\left(\max-\min\right)}{6}$ with the an assumption that the original data followed a normal distribution where median equals the mean; ①Overall efficacy rate; ②Swanson, Nolan, and Pelham Rating Scale, Version IV (SNAP-IV) score; ③Incidence of adverse drug reaction (ADR); ④Electroencephalogram (EEG).

#### 3.2.1 Comparisons

Additionally, among the 21 included studies, there were 9 comparisons in RCTs (viz. Auricular therapy vs. Placebo, CMs+Auricular therapy vs. CMs, Acupuncture+Auricular therapy vs. Synthetic drugs, CMs+Auricular therapy vs. Synthetic drugs, CMs+Synthetic drugs+Acupuncture+Auricular therapy+Acupoint Herbal Patches+Other non-drug therapies vs. CMs+Synthetic drugs, Synthetic drugs+Ear scraping+Auricular therapy vs. Synthetic drugs, Acupuncture+Auricular therapy vs. CMs, CMs+Auricular therapy+Acupuncture vs. CMs+Synthetic drugs, Tuina+Auricular therapy vs. Synthetic drugs) and 4 comparisons in NRCT [viz. CMs+Auricular therapy vs. Synthetic drugs, CMs+Auricular therapy vs. Synthetic drugs, Acupuncture+Auricular therapy+Other non-drug therapies vs. Synthetic drugs, Acupuncture (Plum Blossom Needle) + Auricular therapy vs. CMs].

#### 3.2.2 Main TCM interventions

Firstly, 20 of 21 studies was conducted ear acupressure with seeds, 2 of whom added electroacupuncture stimulation and ear copper bianstone scraping respectively (15, 33), while 1 studies left used ear acupuncture therapy (23). Besides, ear acupoints involved in auricular therapy were reported in 21 studies, including Shen-Men (TF_4_), Jiao-Gan (AH_6a_), Subcortex (AT4), Endocrine (CO18), Heart (CO_15_), Kidney (CO_10_), Spleen (CO_13_), Liver (CO_12_), Gallbladder (CO_11_), Brain Stem (AT_3,4i_), Central Rim (AT_2,3,4i_), Adrenal Gland (TG_2P_), Triple Energizer (CO_17_), Point Zero, Laterality point, Master oscillation point, Temple (AT_2_), Master Cerebral (AT_1_).

Secondly, the acupoints mentioned in included studies contained Bai-Hui (GV20), Shen-Men (HT7), Tai-Chong (LR3), Si-Shen-Cong (EX-HN1), Xin-Shu (BL15), Pi-Shu (BL20), Shen-Shu (BL23), Ding-Shen [0.5 inches above Yintang (GV24^+^) and Yangbai (GB14) acupoints respectively], Nei-Guan (PC6), He-Gu (LI4), Xing-Jian (LR2), Shao-Fu (HT8), Tai-Xi (KI3), San-Yin-Jiao(SP6), Zu-San-Li (ST36), Shuai-Gu (GB8), Nao-Hu (GV17), Shen-Ting (GV24), Feng-Chi (GB20).

Thirdly, 12 of 21 studies were reported to utilize Chinese medicine, including 4 Granules, 4CM-Decoctions, 2 CM soft extracts, 2 Chinese patent medicines, among which 6 were customized CM-preparations, 5 were supplementary formula of Liu-Wei-Di-Huang (20, 24, 31, 34, 36). And detailed constituents of all CMs involved in the included studies were shown in Table 3.

**Table 3:** CM-preparations in included studies.

Category	Item	Ingredients
CM-granules	Supplementary formula of Huan-Gan-Li-Pi Granules	CINNAMOMI RAMULUS, CODONOPSIS RADIX, PORIA, PAEONIAE ALBA RADIX TOSTA, ATRACTYLODIS MACROCEPHALAE RHIZOMA TOSTUM CUM MELLE ET FURFURE, ATRACTYLODIS MACROCEPHALAE RHIZOMA, CITRI RETICULATAE PERICARPIUM, DIOSCOREAE RHIZOMA, LABLAB SEMEN ALBUM TOSTA, GLYCYRRHIZAE RADIX ET RHIZOMA PRAEPARATA CUM MELLE, PORIA CUM PINI RADIX, ACORI TATARINOWII RHIZOMA, ZINGIBERIS RHIZOMA RECENS, JUJUBAE FRUCTUS
	Ningxin Granules	ANEMARRHENAE RHIZOMA, REHMANNIAE RADIX PRAEPARATA, CORNI FRUCTUS, DIOSCOREAE RHIZOMA, PORIA CUM PINI RADIXMOUTAN CORTEX, MARGARITIFERA CONCHA, OPHIOPOGONIS RADIX, HALIOTIDIS CONCHA, UNCARIAE RAMULUS CUM UNCIS, PHELLODENDRI CHINENSIS CORTEX, PAEONIAE ALBA RADIX, LYCII FRUCTUS, CHRYSANTHEMI FLOS, GASTRODIAE RHIZOMA, GLYCYRRHIZAE RADIX ET RHIZOMA
	Customized CM-preparations 1	BUPLEURI RADIX PRAEPARATA CUM ACETO，CURCUMAE RADIX, SCUTELLARIAE RADIX, REHMANNIAE RADIX, UNCARIAE RAMULUS CUM UNCIS, PAEONIAE RADIX ALBA, ACORI TATARINOWII RHIZOMA, MAGNETITUM, ZIZIPHI SPINOSAE SEMEN TOSTUM, ALPINIAE OXYPHYLLAE FRUCTUS, NELUMBINIS PLUMULA
	Customized CM-preparations 2	ACORI TATARINOWII RHIZOMA, MARGARITIFERA CONCHA, PORIA, POLYGALAE RADIX, CURCUMAE RADIX, UNCARIAE RAMULUS CUM UNCIS, ARISAEMA CUM BILE, AURANTII FRUCTUS IMMATURUS, GASTRODIAE RHIZOMA
CM soft extract	Customized CM-preparations 5	MARGARITIFERA CONCHA, ACORI TATARINOWII RHIZOMA, PORIA, POLYGALAE RADIX, CURCUMAE RADIX, UNCARIAE RAMULUS CUM UNCIS, GASTRODIAE RHIZOMA, AURANTII FRUCTUS IMMATURUS, ARISAEMA CUM BILE
	Customized CM-preparations 6	PORIA, MARGARITIFERA CONCHA, ACORI TATARINOWII RHIZOMA, CURCUMAE RADIX, POLYGALAE RADIX, UNCARIAE RAMULUS CUM UNCIS, ARISAEMA CUM BILE, GASTRODIAE RHIZOMA, AURANTII FRUCTUS IMMATURUS
CM-decoction	Supplementary formula of Liu-Wei-Di-Huang and Gan-Mai-Da-Zao decoction	REHMANNIAE RADIX PRAEPARATA, DIOSCOREAE RHIZOMA, CORNI FRUCTUS, MOUTAN CORTEX, PORIA, ALISMATIS RHIZOMA, TRITICI AESTIVI FRUCTUS, JUJUBAE FRUCTUS, GLYCYRRHIZAE RADIX ET RHIZOMA
	Tiaoxie Yizhi decoction	BUPLEURI RADIX, GINSENG RADIX ET RHIZOMA, REHMANNIAE RADIX, POLYGALAE RADIX, EPIMEDII FOLIUM, COPTIDIS RHIZOMA, BAMBUSAE CAULIS IN TAENIAS, LIGUSTRI LUCIDI FRUCTUS
	Customized CM-preparations 3	ASTRAGALI RADIX, CODONOPSIS RADIX, ATRACTYLODIS MACROCEPHALAE RHIZOMA, PORIA CUM PINI RADIX, POLYGALAE RADIX PRAEPARATA CUM MELLE, ZIZIPHI SPINOSAE SEMEN, ANGELICAE SINENSIS RADIX, AUCKLANDIAE RADIX, GLYCYRRHIZAE RADIX ET RHIZOMA
	Customized CM-preparations 4	PORIA, DIOSCOREAE RHIZOMA, REHMANNIAE RADIX PRAEPARATA, CORNI FRUCTUS, ACORI TATARINOWII RHIZOMA, POLYGALAE RADIX, FRUCTUS TRIBULI, DRACONIS OS, CRASSOSTREAE CONCHA, UNCARIAE RAMULUS CUM UNCIS, BOMBYX BATRYTICATUS, COICIS SEMEN, JUJUBAE FRUCTUS, GLYCYRRHIZAE RADIX ET RHIZOMA
Chinese patent medicine	Qi-Ju-Di-Huang pills	REHMANNIAE RADIX PRAEPARATA, CORNI FRUCTUS, DIOSCOREAE RHIZOMA, ALISMATIS RHIZOMA, MOUTAN CORTEX, PORIA, LYCII FRUCTUS, CHRYSANTHEMI FLOS
	Zhi-Bai-Di-Huang pills	REHMANNIAE RADIX PRAEPARATA, CORNI FRUCTUS, DIOSCOREAE RHIZOMA, ALISMATIS RHIZOMA, MOUTAN CORTEX, PORIA, ANEMARRHENAE RHIZOMA, PHELLODENDRI CHINENSIS CORTEX

#### 3.2.3 Outcomes

There were 17 indicators in included RCTs, namely, sleep quality score (1 of 17, 5.88%) (19); Child Behavior Checklist (CBCL) score (3 of 17, 17.64%) (18, 20, 31); Conners Parent Rating Scale (CPRS) score (4 of 17, 23.52%) (15, 16, 21, 33); Children's Somatization Inventory version Ⅳ (CSI-4) score (1 of 17, 5.88%) (15); Conners Index of Hyperactivity (CIH) (5 of 17, 29.41%) (17, 20, 24, 31, 33); overall efficacy rate (13 of 17, 76.47%) (14, 16, 17, 21–26, 30–33); Swanson, Nolan and Pelham Rating Scale, Version Ⅳ (SNAP-Ⅳ) score (2 of 21, 11.76%) (17, 33); Hamilton Anxiety Scale (HAMA) score (2 of 17, 11.76%) (16, 21); Hamilton Depression Scale (HAMD) score (2 of 17, 11.76%) (16, 21); Quality of Life (QoL) Questionnaire score (2 of 17, 11.76%) (16, 21); incidence of adverse drug reaction (ADR) (5 of 17, 23.52%) (15, 21, 23, 24, 30); recurrence rate (2 of 17, 11.76%) (25, 26); Digit Cancellation Test (DCT) score (1 of 17, 5.88%) (24); Raven's Standard Progressive Matrices (SPM) (2 of 17, 11.76%) (20, 31); Comprehensive attention quotient (CAQ) (1 of 17, 5.88%) (33); Wechsler Intelligence Scale for Children (WIC) score (1 of 17, 5.88%) (33); Comprehensive response control quotient (CRCQ) (1 of 17, 5.88%) (33). And the before-and difference of CPRS score, HAMA score, QoL Questionnaire score were calculated for further meta-analyses (Table 4).

**Table 4:** Calculation of scoring in included studies.

Outcome		Study ID	Group	Score (Mean ± SD)		
				Pre-treatment	Post-treatment	Difference^a^^,^^b^
Conners Parent Rating Scale (CPRS) score	Learning Problems	Hong HUANG 2025 (16)	T	1.72 ± 0.55	0.31 ± 0.08	−1.41 ± 0.51
			C	1.73 ± 0.56	0.88 ± 0.12	−0.85 ± 0.51
		Yanhui ZHANG 2023 (21)	T	1.71 ± 0.23	0.38 ± 0.11	−1.33 ± 0.20
			C	1.69 ± 0.22	0.87 ± 0.19	−0.82 ± 0.21
	Psychosomatic Problems	Hong HUANG 2025 (16)	T	1.81 ± 0.03	0.91 ± 0.12	−0.90 ± 0.11
			C	1.82 ± 0.05	1.33 ± 0.06	−0.49 ± 0.06
		Yanhui ZHANG 2023 (21)	T	1.80 ± 0.34	0.92 ± 0.23	−0.88 ± 0.30
			C	1.78 ± 0.29	1.31 ± 0.45	−0.47 ± 0.40
	Conduct Problems	Hong HUANG 2025 (16)	T	1.26 ± 0.33	0.62 ± 0.08	−0.64 ± 0.30
			C	1.27 ± 0.35	0.99 ± 0.12	−0.28 ± 0.31
		Yanhui ZHANG 2023 (21)	T	1.25 ± 0.31	0.63 ± 0.21	−0.62 ± 0.27
			C	1.26 ± 0.28	0.98 ± 0.44	−0.28 ± 0.39
	Impulsivity-Hyperactivity	Hong HUANG 2025 (16)	T	1.70 ± 0.08	0.51 ± 0.03	−1.19 ± 0.07
			C	1.71 ± 0.09	1.05 ± 0.22	−0.66 ± 0.19
		Yanhui ZHANG 2023 (21)	T	1.70 ± 0.52	0.52 ± 0.11	−1.18 ± 0.47
			C	1.67 ± 0.43	1.03 ± 0.15	−0.64 ± 0.38

Outcome		Study ID	Group	Score (Mean ± SD)		
				Pre-treatment	Post-treatment	Difference^a^^,^^b^
Hamilton Anxiety Scale (HAMA) score		Hong HUANG 2025 (16)	T	33.25 ± 2.88	13.61 ± 1.41	−19.64 ± 2.49
			C	33.26 ± 2.89	19.52 ± 2.01	−13.74 ± 2.57
		Yanhui ZHANG 2023 (21)	T	33.21 ± 6.38	13.62 ± 4.09	−19.59 ± 5.56
			C	32.98 ± 5.40	19.51 ± 5.27	−13.47 ± 5.34
Hamilton Depression Scale (HAMD) score		Hong HUANG 2025 (16)	T	32.11 ± 2.11	12.11 ± 1.16	−20.00 ± 1.83
			C	32.12 ± 2.15	17.55 ± 1.96	−14.57 ± 2.06
		Yanhui ZHANG 2023 (21)	T	32.09 ± 5.44	12.16 ± 3.92	−19.93 ± 4.86
			C	31.97 ± 6.30	17.62 ± 4.38	−14.35 ± 5.59
Quality of Life (QoL) Questionnaire score	Physical Functioning (PF)	Hong HUANG 2025 (16)	T	74.22 ± 2.33	88.63 ± 3.11	14.41 ± 2.80
			C	74.25 ± 2.35	80.02 ± 2.88	5.77 ± 2.65
		Yanhui ZHANG 2023 (21)	T	74.15 ± 6.32	88.92 ± 5.44	14.77 ± 5.93
			C	75.08 ± 7.19	81.05 ± 4.39	5.97 ± 6.28
	Social Functioning (SF)	Hong HUANG 2025 (16)	T	72.63 ± 2.11	87.63 ± 3.01	15.00 ± 2.68
			C	72.65 ± 2.12	79.25 ± 2.01	6.60 ± 2.07
		Yanhui ZHANG 2023 (21)	T	72.09 ± 7.12	87.69 ± 4.13	15.60 ± 6.19
			C	71.98 ± 5.90	79.31 ± 6.39	7.33 ± 6.16

Outcome		Study ID	Group	Score (Mean ± SD)		
				Pre-treatment	Post-treatment	Difference^a^^,^^b^
Quality of Life (QoL) Questionnaire score	Role-Physical (RP)	Hong HUANG 2025 (16)	T	69.22 ± 2.11	88.63 ± 2.71	19.41 ± 2.46
			C	69.25 ± 2.12	81.02 ± 2.00	11.77 ± 2.06
		Yanhui ZHANG 2023 (21)	T	69.60 ± 5.21	88.41 ± 6.32	18.81 ± 5.84
			C	70.26 ± 4.92	81.39 ± 5.97	11.13 ± 5.52
	Mental Health (MH)	Hong HUANG 2025 (16)	T	73.22 ± 2.88	89.63 ± 2.71	16.41 ± 2.80
			C	73.25 ± 2.89	82.33 ± 2.16	9.08 ± 2.60
		Yanhui ZHANG 2023 (21)	T	73.36 ± 8.52	89.61 ± 5.78	16.25 ± 7.53
			C	73.45 ± 9.03	83.47 ± 6.37	10.02 ± 8.03

a Mean difference (MD) = *μ* (post-treatment)- *μ* (pre-treatment).

b With the assumption that Pearson coefficient (*r*) equals to 0.5, the difference of SD is calculated (DSD) as DSD = $\surd \left(SD_{pre}^{2}+SD_{post}^{2}-2rSD_{pre}SD_{post}\right)$.

There were 4 indicators in included NRCTs, namely, overall efficacy rate (4 of 4, 100%) (34–37), SNAP-IV score (1 of 4, 25%) (37), ADR (1 of 4, 25%) (37), Electroencephalogram (EEG) (1 of 4, 25%) (34).

### 3.3 Methodological quality of included studies

**Figure 2:**
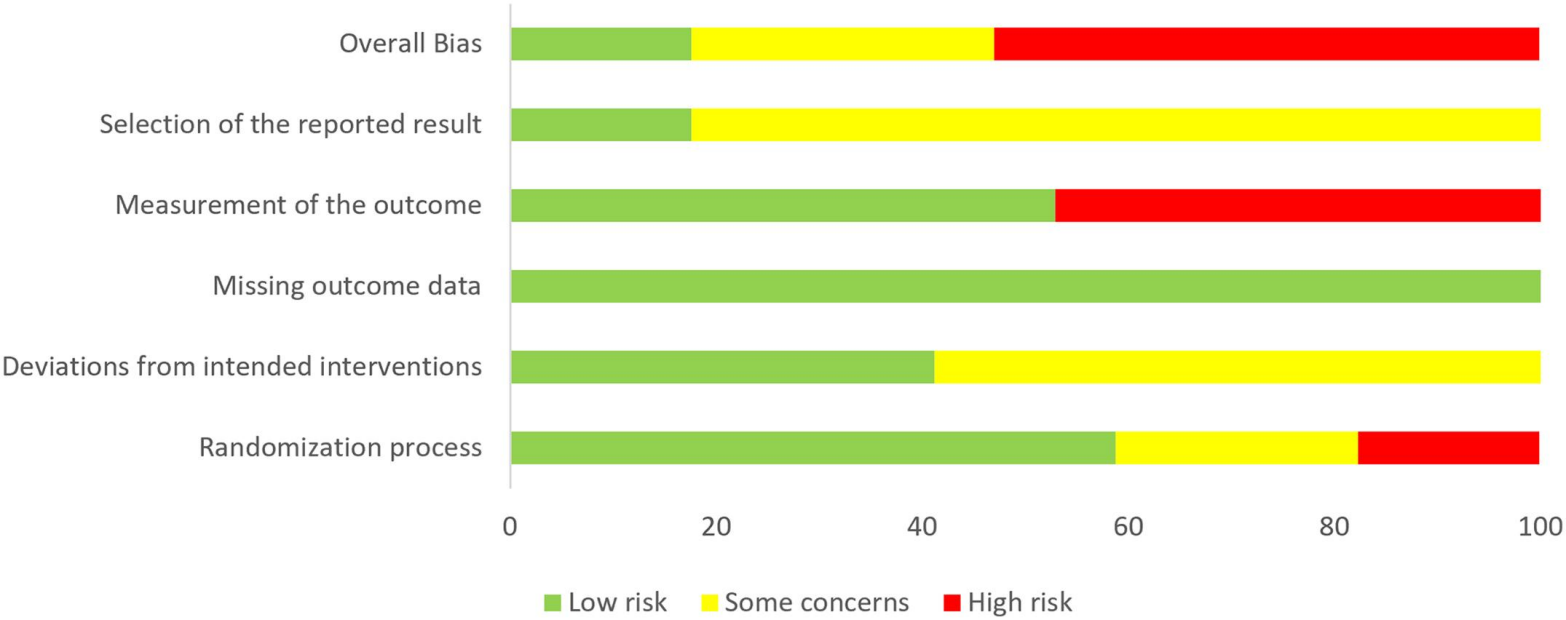
Risk of bias graph of included 17 RCTs (summary graph).

We appraised the 17 RCTs as followed (Figure 2 and Supplementary S1). Firstly, in terms of random sequence generation, 5 studies (29.41%) utilized random number tables (14, 16, 17, 23, 33), 4 studies (23.52%) employed computer-generated randomization (18, 19, 21, 30), 1 study (5.88%) combined random number tables with visit sequence allocation 1 (32), 1 study (5.88%) applied a randomization with ratio of 1:1 (31), 1 study (5.88%) implemented an alternate single/double-day allocation (22) and 1 study (5.88%) utilized sealed opaque envelope method (15), while the remaining 4 studies (23.52%) did not report any specific method for randomization (20, 24–26); Secondly, in terms of allocation concealment, 5 studies (29.41%) had allocation concealment (15, 18, 19, 21, 30), 5 studies (29.41%) may have allocation concealment (14, 16, 17, 23, 33), 3 studies (17.64%) did not have allocation concealment (22, 31, 32), and the remaining 4 studies (23.52%) lost relevant information (20, 24–26); Thirdly, in terms of deviations from intended interventions, 1 study (5.88%) was double-blind (18), 1 study (5.88%) was single blind (15), and the remaining 15 studies (88.23%) did not mention information about blinding methods that were judged as unclear risks (14, 16, 17, 19–26, 30–33); Fourthly, in terms of missing outcome data, all studies (100%) were evaluated as having low risk of bias due to complete outcome data or adequately handled incomplete outcome data (14–26, 30–33); Fifthly, in terms of measurement of the outcome, the methods used to measure results were appropriate in all studies (100%), with the same method for detecting outcomes in both the intervention and the control group; Sixthly, except for 2 studies (11.76%) (18, 19), the other studies (88.24%) assessors may be aware of the specific intervention received by participants. Seventhly, in terms of selection of reported results, 3 studies (17.64%) registered their research protocols and reported registration information (15, 18, 19), while the other 14 studies (82.36%) did not provide data reporting registration information; Finally, all studies (100%) reported comparability of baseline data, therefore were assessed as low risk of other biases (14–26, 30–33).

**Figure 3:**
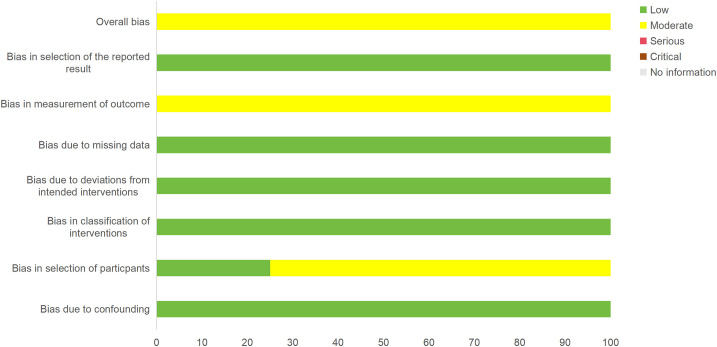
Risk of bias graph of included 4 NRCTs (summary graph).

We appraised the 4 NRCTs as followed (Figure 3 and Supplementary S2). Firstly, all studies have controlled confounding factors such as age and gender so as to be considered as low-risk confounding bias; Secondly, 3 studies included only participants who completed the entire process in line with the therapeutic schedule, resulting in a medium risk of selection bias (34–36); Thirdly, the measure interventions bias is considered as low risk because all 4 studies were retrospective observational studies, where all interventions were known for researchers; Fourthly, all participants adhered to pre-set interventions without additional therapies, which obtained low risk in bias due to deviations from intended interventions; Fifthly, 1 study had a similar proportion of lost to follow-up/incomplete treatment in treatment group compared to the control group (37), while other 3 studies (34–36) did not suffer from missing data, resulting in low-risk bias due to missing data; Sixthly, 4 studies used the same measurement methods for both the treatment group and the control group, and the outcome assessors were aware of the interventions. Accordingly, bias in measurement of outcome were rated as medium risk; Seventhly, none of results were found to be selectively reported, leading to low-risk bias in selection of the reported result.

### 3.4 Analyses of auricular therapy in RCTs

#### 3.4.1 Overall efficacy rate

A total of 13 of 17 studies (76.47%) reported overall efficacy rate, among which the following 8 studies (61.54%) (14, 16, 21, 22, 30–33) yielded significantly better outcomes (*P* < 0.05) compared to different control groups. First, the combination of acupuncture and auricular therapy demonstrated superior efficacy compared with synthetic drugs; And the combination of CMs and auricular therapy was more significantly effective than monotherapy of CMs; Besides, superior outcomes were observed with combination of synthetic drugs, ear scraping and auricular therapy relative to synthetic drugs; Afterwards, the integrated acupuncture and auricular therapy was superior to CMs; In the meantime, the combination of CMs, auricular therapy and acupuncture outperformed the combination of CMs and synthetic drugs. Whereas, 3 of 13 studies (23.08%) (17, 23, 24) did not yield significantly better outcomes, compared to different control groups, in terms of overall efficacy rate, which indicated a tendency that the combination of CMs and auricular therapy was more effective than CMs, and the combination of CMs and auricular therapy was superior to synthetic drugs. On the contrary, 2 of 13 studies (15.38%) (25, 26) reported that the combination of acupuncture and auricular therapy was inferior to synthetic drugs, and the combination of Tuina and auricular therapy was inferior to synthetic drugs (*P* > 0.05).

**Figure 4:**
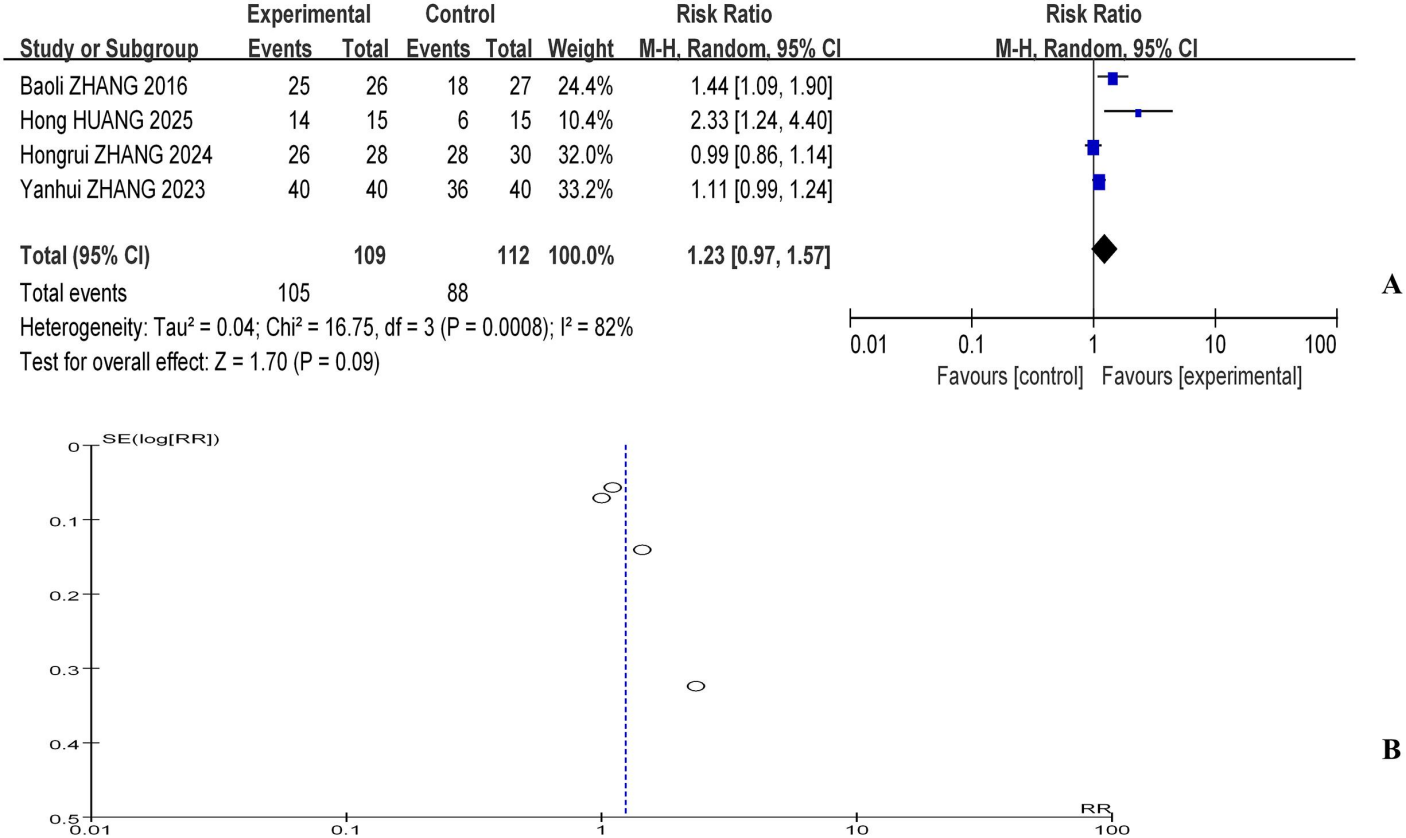
Forest plot of overall efficacy rate: auricular therapy+CMs vs. CMs **(A)** Forest plot; **(B)** funnel plot).

Contrary to expectations, there were 4 of 13 studies (30.76%) that made the comparison—"integrated auricular therapy into CMs vs. monotherapy of CMs”, regarding overall efficacy rate, but 3 of 4 studies (75.00%) (14, 16, 21) reported positive results (*P* < 0.05) while the one left (17) reported no difference in between-group comparison (*P* > 0.05). In order to further figure out this contradictory issue, we conducted meta-analyses among studies above. And statistical significance was not found in the result of meta-analysis (*n* = 221, *RR* = 1.23, 95%*CI*:0.97–1.57, *P* = 0.09, *I^2^* = 82%) (Figure 4A). Then, leave-one-out method was applied to perform sensitivity analysis, and statistically significant before-and-after difference was not observed (Supplementary Figure S3). However, when one of the studies (16) was removed, the *I^2^* dropped from 82% to 70%, which suggested that this study was a potential source of heterogeneity. Due to limited studies, obvious publication bias was not detected in meta-analyses with small-study effects by funnel plot and Harbord test (T-score test) (38, 39) (Figure 4B, Supplementary Table S3-S5). And the meta-analyses with fewer than 4 studies would not be executed Harbord test.

**Figure 5:**
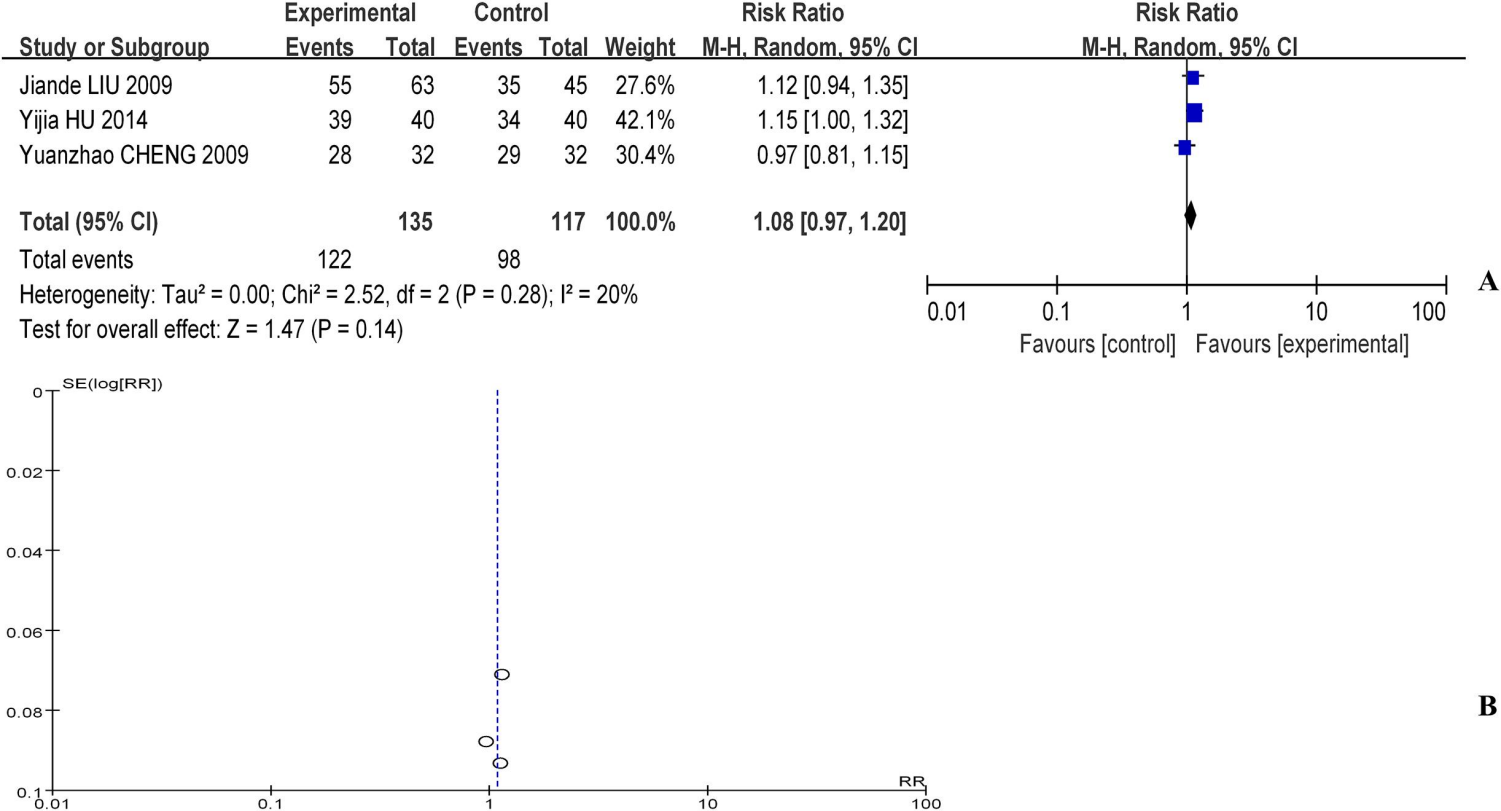
Forest plot and funnel plot of overall efficacy rate: acupuncture + auricular therapy vs. synthetic drugs **(A)** Forest plot; **(B)** funnel plot.

Similarly, another 3 of 13 studies (22, 25, 30) (23.07%) made the comparison—"acupuncture + auricular therapy vs. synthetic drugs”, but 2 of 3 studies (22, 30) reported positive results (*P* < 0.05) while no statistical significance was found in the one left (25) (*P* > 0.05). Thus, we obtained a negative result (*n* = 252, *RR* = 1.08, 95% *CI*:0.97–1.20, *P* = 0.14, *I^2^* = 20%) by meta-analysis (Figure 5). Then, leave-one-out method was applied to perform sensitivity analysis, and statistically before-after difference was not observed (Supplementary Figure S4).

Finally, another 2 of 13 studies (23, 24) (15.38%) that made the comparison—"CMs+auricular therapy vs. synthetic drugs”, which did not indicated statistically significant difference in the total effective rate between the treatment and control groups. And we also obtained a negative result (*n* = 118, *RR* = 1.06, 95% CI:0.89–1.26, *P* = 0.50 *I^2^* = 0%) by meta-analysis (Supplementary Figure S5).

#### 3.4.2 CPRS score

A total of 4 of 17 studies (15, 16, 21, 33) (23.52%) reported CPRS score, and they significantly yielded better outcomes compared to different control groups (*P* < 0.05). First, the auricular therapy demonstrated superior efficacy compared with placebo. Besides, combination of CMs and auricular therapy was more significantly effective than CMs. And combination of drugs, ear scraping and auricular therapy was superior to synthetic drugs.

Among 4 studies, 2 of them (50%) (16, 21) made the same comparison—"CMs + auricular therapy vs. CMs”, with positive results (*P* < 0.05) in 4 dimensions (learning problems, psychosomatic problems, conduct problems and impulsivity-hyperactivity). And we also obtained the same result via meta-analyses (Supplementary Figure S6).

#### 3.4.3 HAMA score and HAMD score

A total of 2 of 17 studies (16, 21) (11.76%) reported HAMA score and HAMD score, and they significantly yielded better outcomes compared to different control groups (*P* < 0.05). The combination of CMs and auricular therapy demonstrated superior efficacy compared with CMs. And the result of meta-analysis (Supplementary Figure S7) remained the same.

#### 3.4.4 Qol questionnaire score

A total of 2 studies of 17 (16, 21) (11.76%) reported QoL Questionnaire score, with positive results (*P* < 0.05) in 4 dimensions (Social functioning, Physical functioning, Role-physical domain, Mental health), compared to control groups. And we also found that the combination of CMs and auricular therapy demonstrated superior efficacy compared with CMs, via meta-analyses (Supplementary Figure S8).

#### 3.4.5 Incidence of ADR

There were 5 of 17 studies (15, 21, 23, 24, 30) (29.41%) reported ADRs. Firstly, 2 of 5 studies (40%) (24, 30) reported that the incidence of ADRs in the treatment group was lower than that in the control group (*P* < 0.05). Concretely, decreased medication risk was demonstrated in 2 comparisons (viz., acupuncture+auricular therapy vs. synthetic drugs, CMs + auricular therapy vs. synthetic drugs). Besides, 1 of 5 studies (20%) (21) suggested none of statistically significant difference of ADRs in the comparison (CMs + auricular therapy vs. CMs), while another 1 of 5 studies (20%) (23) reported none of ADR in treatment group and did not mention the data in control group (CMs+auricular therapy vs. synthetic drugs). Whereas, only 1 of 5 studies (20%) (15) did not described presence of ADR in the comparison (auricular therapy vs. placebo) between 2 groups.

#### 3.4.6 Other outcomes

From our findings (Table 5), auricular therapy combined with other treatments was benefit for ADHD kids in improving sleep quality (19), psychological and behavioral problems (viz., CBCL score, CSI-4 score, CIH score, CRCQ, SNAP-IV score) (15, 17, 18, 20, 24, 31, 33), level of intelligence and attention (viz., WIC, DCT, CAQ, CRCQ, SPM) (20, 24, 31, 33), as well as in reducing recurrence rate (25, 26). However, 1 of 17 studies (5.88%) reported no statistically significant improvement in CIH score between two groups (CMs+auricular therapy vs. synthetic drugs) (24), while another study (5.88%) found that the statistical difference (CMs+auricular therapy vs. CMs) in improvement depended on intervention duration (*P* < 0.05 in 4th week and 6th week 6; *P* > 0.05 in 2nd week) (31).

**Table 5:** Summary of other outcomes in 17 included RCTs.

Outcome	Observed period	Comparison		Authors’ conclusion
		Superior group	Inferior group	
Sleep quality score (19)	1 month	Auricular therapy	Placebo	a
CBCL score (18)	8 weeks	Auricular therapy	Placebo	a
CBCL score (20)	4 weeks	CMs + Synthetic drugs + Acupuncture + Auricular therapy + Acupoint Herbal Patches + Other non-drug therapies	CMs + Synthetic drugs	a
CBCL score (31)	2 months	CMs + Auricular therapy + Acupuncture	CMs + Synthetic drugs	a
CSI-4 score (15)	8 weeks	Auricular therapy	Placebo	a
CIH score (33)	2 months	Synthetic drugs + Ear scraping + Auricular therapy	Synthetic drugs	a
CIH score (17)	6 weeks	CMs + Auricular therapy	CMs	a
CIH score (20)	4 weeks	CMs + Synthetic drugs + Acupuncture + Auricular therapy + Acupoint Herbal Patches + Other non-drug therapies	CMs + Synthetic drugs	a
CIH score (31)	2 months	CMs + Auricular therapy + Acupuncture	CMs + Synthetic drugs	a
CIH score (24)	8 weeks	CMs + Auricular therapy	Synthetic drugs	b
SNAP-Ⅳ score (33)	2 months	Synthetic drugs + Ear scraping + Auricular therapy	Synthetic drugs	a
SNAP-Ⅳ score (17)	2 weeks	CMs + Auricular therapy	CMs	b
	4 weeks			a
	6 weeks			a
Recurrence rate (25)	3 months	Acupuncture + Auricular therapy	Synthetic drugs	a
Recurrence rate (26)	1 month	Tuina + Auricular therapy	Synthetic drugs	a
DCT score (24)	8 weeks	CMs + Auricular therapy	Synthetic drugs	a
SPM score (20)	4 weeks	CMs + Synthetic drugs + Acupuncture + Auricular therapy + Acupoint Herbal Patches + Other non-drug therapies	CMs + Synthetic drugs	a
SPM score (31)	2 months	CMs + Auricular therapy + Acupuncture	CMs + Synthetic drugs	a
CAQ score (33)	2 months	Synthetic drugs + Ear scraping + Auricular therapy	Synthetic drugs	a
WIC score (33)	2 months	Synthetic drugs + Ear scraping + Auricular therapy	Synthetic drugs	a
CRCQ score (33)	2 months	Synthetic drugs + Ear scraping + Auricular therapy	Synthetic drugs	a

a, positive conclusion with statistical significance; b, positive conclusion without statistical significance;.

### 3.5 Analyses of auricular therapy in NRCTs

#### 3.5.1 Overall efficacy rate

All 4 included NRCTs reported the overall efficacy rate, with a consistent conclusion that it performed better in the treatment group than that in the control group (*P* < 0.05) (34–37), which suggested that auricular therapy could play a positive role in treating ADHD. Concretely, the combination of CMs and auricular therapy was superior to synthetic drugs, the combination of acupuncture, auricular therapy as well as other non-drug therapies was superior to synthetic drugs, and the combination of acupuncture and auricular therapy was superior to CMs.

**Figure 6:**
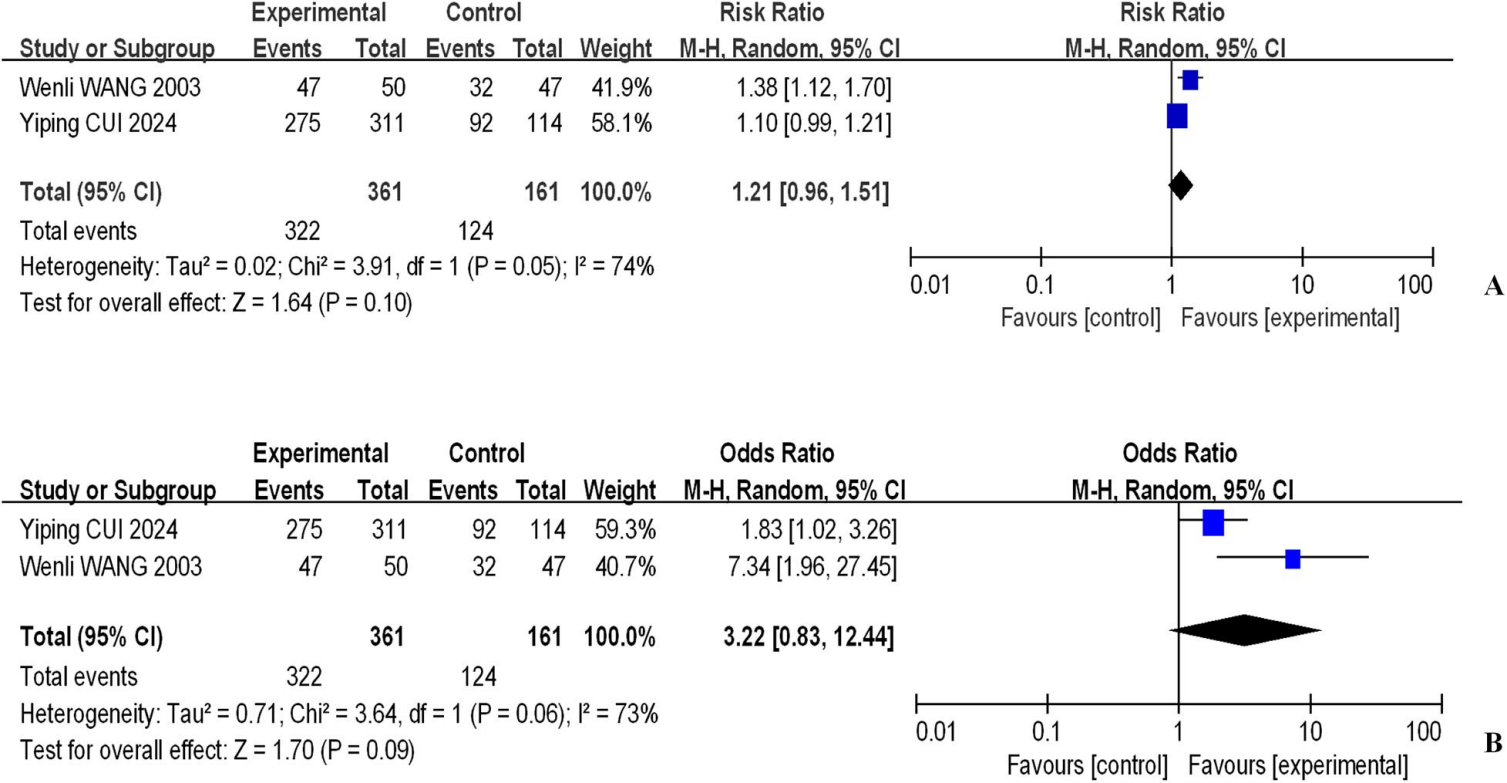
Forest plot of overall efficacy rate in 2 NRCTs: CM + auricular therapy vs. synthetic drugs **(A)** Risk Ratio; **(B)** Odds Ratio.

A total of 2 studies (50.00%) made the same comparison—"CM + Auricular therapy vs. Synthetic drugs" (36, 37), we also obtained the same result via meta-analyses but statistical significance was not observed (*n* = 522, *RR* = 1.21, 95%*CI*:0.96–1.51, *P* = 0.1, *I^2^* = 74%) (Figure 6).

#### 3.5.2 Other outcomes

SNAP-IV score and incidence of ADRs were reported in one study (37), while EEG results were reported in a separate study (34). We found significant reduction in SNAP-IV scores (*P* < 0.05) in the treatment group compared to synthetic drugs, which suggested that combination of CMs and auricular therapy was beneficial to ADHD. And the results indicated that the combination of acupuncture and auricular therapy was superior to CMs in improving abnormal EEG (*P* < 0.05). Meanwhile, the incidence of ADRs in the treatment group was significantly lower than in the control group (*P* < 0.05), which revealed that the combination of CMs and auricular therapy was superior to synthetic drugs.

## 4 Discussion

### 4.1 Main findings and implications for future work

This review contained 21 studies involving 2,270 participants from 17 RCTs and 4 NRCTs, for exploring the adjunctive effectiveness of auricular therapy in ADHD treatment. On the one hand, the 17 RCTs with a few low-certainty evidence (Figure 2) have shown effective treatment of adjuvant auricular therapy in alleviating behavioral disorders, impulsive and hyperactive symptoms, as well as in reducing anxiety and depression in children via meta-analysis. Besides, we found an effective tendency for improving overall efficacy rate and decreasing incidence of ADRs via meta-analysis. And other descriptive evidence showed that adjunctive auricular therapy was beneficial to sleep quality, social competence, recurrence rate, level of intelligence and attention. Moreover, a study (17) suggested that the efficiency of long-term auricular therapy may be more measurable in improving ADHD-related outcomes; One the other, the 4 NRCTs with low-and-moderate-certainty evidence (Figure 3) suggested that the auricular therapy combined with other interventions could significantly improve overall efficacy rate, symptoms of impulsivity-hyperactivity, EEG abnormalities, and could reduce the incidence of ADRs.

Dramatically, despite most RCT studies (3 of 4) (14, 16, 21) suggesting that auricular therapy exhibited advantages in treating pediatric ADHD (*P* < 0.05), the meta-analysis revealed a negative pooling result of overall efficacy rate (Figure 4A, Supplementary Figure S3). And this inconsistency can be primarily attributed to 3 factors: (1) high heterogeneity among included studies (*I^2^* = 82%); (2) decreased overall effect size due to another larger-sample study (1 of 4) (17) with a negative result and higher weight; (3) limited statistical power caused by insufficient sample that could not detect a potential small but statistically significant effect size.

### 4.2 Current research gaps

Although some studies (40–42) related to auricular therapy may have provided preliminary clues to clinical practice for pediatric ADHD, our scoping review could identify several gaps in auricular therapy for ADHD treatment. To begin with, the efficacy of auricular therapy is not certain due to insufficient support from high-quality RCTs, although a variety of RCTs involved with combined therapies were published. Subsequently. Subsequently, population of current RCTs ranging from 30 to 108 is limited, caused by high research costs, low efficiency and recruitment difficulties, which may result in restricted generalizability (43). Additionally, Since children with ADHD probably have suboptimal adherence compared to other population, and the medication for pediatric diseases is relatively simple with fewer confounding factors, real-world studies (e.g., pragmatic clinical trials, PCTs) may have stronger clinical operability and applicability (44). Thus, more protocol-driven PCTs are encouraged to supplement current clinical evidence; Afterwards, most current studies were not reported according to the statement of the Unified Standard for Clinical Trial Reports (CONSORT) and the guidelines of the Standard for Intervention Reporting of acupuncture and moxibustion Controlled Trials (STRICTA), which could ensure the transparency and quality of the reported trials. For instance, the 4 moderate-certainty NRCTs failed to eliminate confounding factors from group allocation and outcome assessment (45); Finally, most studies did not report related adverse events of auricular therapy or its combined therapies, and whether auricular therapy could reduce the incidence of ADRs remains unclear.

### 4.3 Limitations

We need to state some limitations in this study. In the first place, there was substantial heterogeneity among the included literature in terms of study design, intervention protocols and outcome indicators, and meta-analysis was only performed on a small number of studies for quantitative synthesis; Additionally, our results may be compromised with published language restrictions, limited samples, high heterogeneity, unstandardized reporting, and other unmentioned or unclear bias in both RCTs and NRCTs. Lastly, since TCM prioritizes individualized treatment over standardized interventions, TCM practitioners may select different auricular points for ADHD management. Accordingly, our study cannot identify a single optimal therapeutic regimen.

## 5 Conclusion

Our study is first to conduct a scoping review embracing RCTs and NRCTs for exploring auricular therapy as adjuvant therapy in pediatric ADHD, which indicated that auricular therapy as an adjuvant therapy could improve ADHD symptoms to some extent, and longer-term interventions are more likely to detect unobvious improvements on relevant outcomes. However, our evidence needs more real-world studies to completement because of existing risk of bias from RCTs and NRCTs.

## Data Availability

The original contributions presented in the study are included in the article/Supplementary Material, further inquiries can be directed to the corresponding author.

## References

[1] Tan J Li X Tang Z Chen Y Chen L. Protocol of clinical practice guideline for the diagnosis, assessment, and treatment of attention-deficit/hyperactivity disorder in children and adolescents in China (2025 edition). J. Pediatr. Pharmacy. (2025) 31(10):1–5.

[2] May T Birch E Chaves K Cranswick N Culnane E Delaney J. The Australian evidence-based clinical practice guideline for attention deficit hyperactivity disorder. Aust N Z J Psychiatry. (2023) 57(8):1101–16. 10.1177/00048674231166329 37254562 PMC10363932

[3] Farokhzadi F Khajevand Khosli A Mohamadi MR Akbarfahimi M Ali Beigi N. Attention-deficit/hyperactivity disorder. Nat Rev. (2015) 1:1–23. 10.1038/nrdp.2015.20

[4] Wang H. Common neurological comorbidity and treatments of ADHD in children. Chin J Pract Pediatr. (2023) 38(8):592–6.

[5] Popit S Serod K Locatelli I Stuhec M. Prevalence of attention-deficit hyperactivity disorder (ADHD): systematic review and meta-analysis. Eur Psychiatry. (2024) 67(1):1–23. 10.1192/j.eurpsy.2024.1786 39381949 PMC11536208

[6] Li S Feng W Fang F Dong X Zhuang Z Yang Q. Prevalence of attention deficit and hyperactivity disorder in children in China: a systematic review and meta-analysis. Chin J Epidemiol. (2018) 39(7):993–8. 10.3760/cma.j.issn.0254-6450.2018.07.024 30060318

[7] Faraone SV Biederman J Mick E. The age-dependent decline of attention deficit hyperactivity disorder: a meta-analysis of follow-up studies. Psychol Med. (2005) 36(2):159–65. 10.1017/s003329170500471x 16420712

[8] Zhao K Li X. Advances in diagnosing adult attention deficit hyperactivity disorder. Zhejiang Med J. (2022) 44(22):2461–5.

[9] Han X. Expert consensus on integrated Chinese and western medicine diagnosis and treatment of attention deficit hyperactivity disorder. China J Trad Chin Med Pharma. (2023) 38(4):1674–9.

[10] Li T. Consensus on pediatric clinical practice of early identification, standardized diagnosis and treatment of attention deficit hyperactivity disorder. Chin J Pediatr. (2020) 58(3):188–93. 10.3760/cma.j.issn.0578-1310.2020.03.006 32135589

[11] Posner J Polanczyk GV Sonuga-Barke E. Attention-deficit hyperactivity disorder. Lancet. (2020) 395(10222):450–62. 10.1016/s0140-6736(19)33004-1 31982036 PMC7880081

[12] Yijun C Xuemei W. Acupuncture combined with auricular acupressure and psychological intervention for the treatment of childhood hyperactivity disorder: therapeutic efficacy. Shenzhen J Integr Trad Chin West Med. (2018) 28(14):52–4.

[13] Van Vyve L Dierckx B Lim CG Danckaerts M Koch BCP Häge A. Pharmacotherapy for ADHD in children and adolescents: a summary and overview of different European guidelines. Eur J Pediatr. (2023) 183(3):1047–56. 10.1007/s00431-023-05370-w 38095716

[14] Zhang B. Effect of Anshen Huatan method combined with erxue maidou method in the treatment of ADHD. Chin Pediatr Integr Trad West Med. (2016) 8(4):447–49.

[15] Binesh M Daghighi MR Shirazi E Oleson T Hashem-Dabaghian F. Comparison of auricular therapy with sham in children with attention deficit/hyperactivity disorder: a randomized controlled trial. J Altern Complement Med. (2020) 26(6):515–20. 10.1089/acm.2019.0477 32434376

[16] Huang H Liu C Xu Y Bao L Ma X. Therapeutic effect of auricular acupressure with Chinese herbal paste on children with ADHD and analysis of adverse emotional improvement. Chinese Science Citation Database. (2025) (1):24–8.

[17] Zhang H. Clinical observation of modified huan-gan-li-spi decoction combined with auricular acupressure in the treatment of attention deficit hyperactivity disorder in children (Master’s thesis). Liaoning University of Traditional Chinese Medicine (2024).

[18] Mahdavi F Asgarian FS Aghajani M. The effect of ear acupressure on behavioral problems in children with attention-deficit/hyperactivity disorder: a randomized clinical trial. Med Acupunct. (2024) 36(2):93–101. 10.1089/acu.2023.0028 38659722 PMC11036156

[19] Mohammadi L Tagharrobi Z Sharifi K Sooki Z Zare M Zare Joshaghani F. The effect of auriculotherapy on sleep quality in children with attention deficit hyperactivity disorder: a randomized clinical trial. BMC Pediatr. 2025 25(1):48. 10.1186/s12887-024-05371-0 39833716 PMC11744890

[20] Cheng S Zhang D Li X Yuan Z Jin Y. Clinical study on the combined therapy of acupuncture and auricular point pressing for children with hyperactivity disorder of liver-kidney yin deficiency type. Special Health Issue. (2024) (2):83–4.

[21] Zhang Y Zhao F. Analysis of the therapeutic effect of auricular acupressure combined with Chinese herbal paste on children with ADHD and improvement of negative emotions. Chin Health Care. (2023) 41(5):21–4.

[22] Hu Y Huang R Qin Z Luo X Zeng Y. Acupuncture combined with ear bean treatment for attention deficit hyperactivity disorder in children. J Clin Acupuncture Moxibustion. (2014) 30(4):15–7.

[23] Xu Y Liu C Miao J Ma X. Clinical study on treatment of attention deficit hyperactivity disorder in children with auricular acupuncture combined with traditional Chinese medicine. Chin J Ethnomed Ethnopharma. (2017) 26(24):85–7.

[24] Zhang Y Qin Y Ouyang X Zhang D Oouyang L. Clinical observation of ningxin decoction combined with auricular seed pressing in the treatment of children with ADHD. Chin J Ethnomed Ethnopharm. (2019) 28(5):68–70.

[25] Cheng Y Cui L Cao Y. Observation of therapeutic effects of acupuncture at acupoints and auricular point pressing in 32 cases of children with hyperactivity disorder. J Med Inform. (2009) 22(10):909–10.

[26] Zhuo Y. Clinical study on tuina and auricular point application in the treatment of ADHD in children. Jilin J Chin Med. (2006) 26(7):41–50.

[27] Tricco AC Lillie E Zarin W O'Brien KK Colquhoun H Levac D. PRISMA Extension for Scoping Reviews (PRISMA-ScR): checklist and explanation. Ann. Intern. Med. (2018) 169(7):467–73. 10.7326/M18-0850 30178033

[28] Nations U. Convention on the Rights of the Child. Part 1: UN. (1989). p. 2.

[29] Zhou R Xia Q Shen H Yang X Zhang Y Xu J. Diagnosis of children’s attention deficit hyperactivity disorder (ADHD) and its association with cytomegalovirus infection with ADHD: a historical review. Int J Clin Exp Med. (2015) 8(8):13969–75. 26550354 PMC4613039

[30] Liu J. Clinical Research on ADHD Treatment with Jin’s Three-needle Therapy (Doctoral Dissertation). Guangzhou University of Chinese Medicine (2009).

[31] He S. The Clinical Study on Treating Attention Defivit Hyperactivity Disoerder due to Yin Deficiency of Liver and Kidney by Acupuncture, Ear-acupoint Application and Chinese Herbs (Doctoral Dissertation). Guangzhou University of Chinese Medicine (2013).

[32] Wu X Wu Y. Clinical study on acupuncture treatment for ADHD with heart-spleen deficiency syndrome. Guangming J Chin Med. (2016) 31(4):541–2.

[33] Zhong Z Zou L Shi X. Efficacy of atomoxetine hydrochloride combined with ear copper bianstone scraping and bean pressing in children with attention-deficit hyperactivity disorder. World J Integr Trad Western Med. (2023) 18(01):179–82.

[34] Hong L. Clinicai study on acupuncture treatment of 380 cases of infantile attentjon-deficit hyperactivity disorder. Shanghai J Acupunct Moxibustion. (2004) 23(08):23–5.

[35] Meiying W Yanmei W Baoli W. Acupuncture combined with auricular point pressure and psychological intervention in the treatment of 38 cases of ADHD in children. Modern J Integrated Trad Chin West Med. (2009) 18(35):4378–425.

[36] Wenli W Hua F. Efficacy of Chinese herbal medicine for nourishing yin and resolving phlegm combined with auricular acupoint pressing in the treatment of 50 cases of ADHD in children. J Trad Chin Med. (2003) 44(8):609.

[37] Yiping C. Analysis of the real world curative effect of the treatment of attention deficit hyperactivity disorder with tiaoxie yizhi decoction combined with ear acupoint pressing beans (Master’s thesis). Shandong University of Traditional Chinese Medicine (2024).

[38] Harbord RM Egger M Sterne JAC. A modified test for small-study effects in meta-analyses of controlled trials with binary endpoints. Stat Med. (2005) 25(20):3443–57. 10.1002/sim.2380 16345038

[39] Lin L. Hybrid test for publication bias in meta-analysis. Stat Methods Med Res. (2020) 29(10):2881–99. 10.1177/0962280220910172 32290810 PMC7434640

[40] Wang M Ma B. Research progress of non-drug treatment of TCM and western medicine for attention deficit hyperactivity disorder. Chinese J Inform TCM. (2023) 30(05):176–81.

[41] Zhang S. Progress in clinical research on external therapy of traditional Chinese medicine (TCM) for children with attention deficit hyperactivity disorder (ADHD). Inner Mongolia J Trad Chin Med. (2023) 42(7):161–3. 10.16040/j.cnki.cn15-1101.2023.07.024

[42] Zhuang T Liu J Cai K Lin H Lai D. Exploration of acupoint selection rules of auricular points for treating children with attention deficit hyperactivity disorder based on data mining. New Chin Med. (2024) 56(12):141–6.

[43] Wei X Lu C Fu J Zhang Y Yang G Xiao W. Construction of an evidence-based scientific research system for prevention and treatment of glycolipid metabolic multimorbidity in integrative Chinese and western medicine from “evidence generation-evidence synthesis-evidence translation” framework. Glob Trad Chin Med. (2026). https://link.cnki.net/urlid/11.5652.R.20260122.1919.016

[44] Hu S. Guideline on real world study design of traditional Chinese medicine for pediatric disease. Drug Evaluation Research. (2023) 46(4):743–51.

[45] Yan W. Lecture 6: reporting guidelines for non-randomized controlled trials — interpretation of the TREND checklist. Chin J Evid Based Pediatr. (2010) 5(6):458–60.

