# Supplementary material for: Auricular therapy as adjunctive treatment for pediatric attention deficit hyperactivity disorder: a scoping review
Source: Front Pediatr. 2026 Jun 26;14:1829733. doi: 10.3389/fped.2026.1829733 (PMC13349770; doi:10.3389/fped.2026.1829733)
Supplement: Supplementary file 1 [file Supplementaryfile1.docx]

Tables

Table S1 Search Strategies

| No. | Database | Search Query | |
| --- | --- | --- | --- |
| 1 | PubMed  (1952.01.01-2025.07.25) | #1 | (((((("Attention Deficit Disorder with Hyperactivity"[MeSH Major Topic]) OR ("ADHD"[Title/Abstract])) OR ("ADDH"[Title/Abstract])) OR ("Attention Deficit Hyperactivity Disorder*"[Title/Abstract])) OR ("Hyperkinetic Syndrome"[Title/Abstract])) OR ("Attention Deficit Disorder*"[Title/Abstract])) OR ("Minimal Brain Dysfunction"[Title/Abstract]) |
|  |  | #2 | ((((((("Auriculotherapy"[Mesh]) OR ("Auricular Therapy"[Title/Abstract])) OR ("Auricular stimulation"[Title/Abstract])) OR ("Auricular Acupuncture*"[Title/Abstract])) OR ("Ear Acupuncture"[Title/Abstract])) OR ("Auricular Acupressure"[Title/Abstract])) OR ("Auricular plaster therapy"[Title/Abstract])) OR ("ear-acupressure"[Title/Abstract]) |
|  |  | #3 | #1 AND #2 |
| 2 | CNKI  (1915.01.01-2025.07.25) | #1 | (SU%='耳穴压豆'+'耳穴埋压'+'耳穴贴压'+'耳穴压籽'+'耳穴疗法'+'耳针'+'耳穴'+'王不留行籽') AND (SU%='注意缺陷多动障碍'+'多动症'+'注意缺陷'+'ADHD'+'ADDH'+'轻微脑功能障碍') |
| 3 | Wanfang  (1910-2025.07.25) | #1 | 主题:("注意缺陷多动障碍"OR"多动症"OR"轻微脑功能障碍"OR"ADDH"OR"ADHD") and 主题:("耳穴"OR"耳针") |
| 4 | VIP  (1989-2025.07.25) | #1 | M=("注意缺陷多动障碍" OR "多动症" OR "注意缺陷" OR "轻微脑功能障碍") AND M=("耳穴" OR "耳针") |
| 5 | Web of science  (1900-2025.07.25) | #1 | TS=("Attention Deficit Disorder with Hyperactivity" or "Attention Deficit Hyperactivity Disorder*" or "Attention Deficit-Hyperactivity Disorder" or "ADHD" or "ADDH" or "Hyperkinetic Syndrome" or "Attention Deficit Disorder*" or "Minimal Brain Dysfunction") |
|  |  | #2 | TS=("Ear Acupuncture"or"Auricular Acupuncture*"or"Auricular Acupressure"or"Ear seed therapy"or"Ear seed application"or"Auricular plaster therapy"or"ear-acupressure"or"Auriculotherap*"or"Auricular Therapy"or"Auricular stimulation"or"Ear reflexology"or"Ear reflex points") |
|  |  | #3 | #1 AND #2 |
| 6 | EBSCO  (1950.01.01-2025.07.25) | #1 | XB ("adhd"or "attention deficit hyperactivity disorder" or "attention deficit disorder" or "attention deficit disorder with hyperactivity") AND XB ("auriculotherapy" or "auricular therapy"or"auricular acupressure"or"auricular point"or"ear acupressure") |

Table S1 Search Strategies (continued)

| No. | Database | Search Query | |
| --- | --- | --- | --- |
| 7 | Embase  (1974-2025.07.25) | #1 | 'attention deficit hyperactivity disorder'/exp AND [embase]/lim |
|  |  | #2 | ((('adhd':ab,ti OR 'attention deficit':ab,ti) AND'disruptive behavior disorders':ab,ti OR 'attention deficit':ab,ti) AND 'disruptive behaviour disorders':ab,ti OR 'attention deficit disorder':ab,ti OR 'attention deficit disorder with hyperactivity':ab,ti OR 'attention deficit hyperactivity disorder':ab,ti) AND [embase]/lim |
|  |  | #3 | #1 OR #2 |
|  |  | #4 | ('auricular acupuncture'/exp OR 'auricular acupuncture') AND [embase]/lim |
|  |  | #5 | ('acupuncture, ear':ab,ti OR 'acupuncture, earlobe':ab,ti OR 'auriculo-acupuncture':ab,ti OR 'auriculoacupuncture':ab,ti OR 'auriculotherapy':ab,ti OR 'ear acupuncture':ab,ti OR 'earlobe acupuncture':ab,ti OR 'auricular acupuncture':ab,ti) AND [embase]/lim |
|  |  | #6 | #4 OR #5 |
|  |  | #7 | #3 AND #6 |

Table S2 List of excluded studies via full-text screening

| No. | Title | Author | Reason |
| --- | --- | --- | --- |
| 1 | Syndrome Differentiation-Based Treatment Primarily Using Auricular Pressure for ADD in Children: A Case Series of 165 Patients | Xia GONG | Study of attention deficit disorder with complications |
| 2 | Clinical Observation of Scalp "Three Needles" Combined with Auricular Therapy as Adjunctive Treatment to Clonidine for Pediatric ADHD | Jing ZHAI | Irrelevant study |
| 3 | Clinical Observation of Ningxin Decoction Combined with Auricular Plaster Therapy for ADHD in Children | Xiaoxiao ZHANG | Study of self-defined criteria for efficacy assessment |
| 4 | Evaluation of Nursing Intervention with Auricular Plaster Therapy in Children with ADHD | Hong HUANG | Study of self-defined criteria for efficacy assessment |
| 5 | Therapeutic Effects of Auricular Seed Pressing Combined with Chinese Herbal Ointment in Children with ADHD: A Clinical Observation | Hongyan XING | Study of self-defined criteria for efficacy assessment |
| 6 | Acupuncture combined with Auricular Application in the Treatment of Infantile Attention-deficit Hyperactivity Disorder for 18 cases | Zhen LI | Study of self-defined criteria for efficacy assessment |
| 7 | Clinical Observation of Auricular Acupressure for MBD in Children: A 40-Case Series | Min LIU | Study of self-defined criteria for efficacy assessment |
| 8 | Early Clinical Study of Pediatric Tuina and Auricular Plaster Therapy for ADHD | Yue ZHUO | Replicate study |
| 9 | Acupuncture plus Auricular Plaster Therapy for MBD in Children: A Case Series of 48 Patients | Li LIU | Study of self-defined criteria for efficacy assessment |

Table S2 List of excluded studies via full-text screening(continued)

| No. | Title | Author | Reason |
| --- | --- | --- | --- |
| 10 | Clinical observations on the treatment of Child Hyperkinetic Sydrome by acupunctrue plus flash cupping | Yulan XI | Study of self-defined criteria for efficacy assessment |
| 11 | Auricular-Body Electroacupuncture Combined with Auricular Press Pellet Therapy for MBD in Children: A Multicenter Case Series of 498 Patients | Liming ZHOU | Study of self-defined criteria for efficacy assessment |
| 12 | Auricular Plaster Therapy Combined with Chinese Herbal Medicine for Hyperkinetic Syndrome in Children: A Case Series of 52 Patients | Junfang LI | Study of self-defined criteria for efficacy assessment |
| 13 | Comprehensive Therapy for MBD in Children: A Report of 204 Cases | Liming ZHOU | Study of self-defined criteria for efficacy assessment and replicate |
| 14 | Clinical Observation on Auricular Pressure for Minimal Brain Dysfunction in 64 Cases | Shijuan YUAN | Study of self-defined criteria for efficacy assessment |
| 15 | Efficacy of Comprehensive TCM Therapy for ADD in Children: An Observation | Da XIAO | Study of self-defined criteria for efficacy assessment |
| 16 | Clinical Study on Acupuncture in Treating Infantile Attention-deficit Hyperactivity Disorder | Hong LI | Replicate study |
| 17 | Therapeutic Efficacy of Acupuncture plus Auricular Plaster Therapy with Psychological Intervention in Children with Attention Deficit Hyperactivity Disorder‌ | Yijun CHEN | Studies where both the experimental group  and control group received auricular therapy |
| 18 | Clinical Observation of Acupuncture in 143 Cases of Pediatric MBD | Yuxiu JI | Studies where both the experimental group  and control group received auricular therapy |
| 19 | The effects observation of acupuncture of regulating yin,yang and five viscera in the treatment of 34 cases of children with intractable attention deficit hyperactivity disorder | Jingmei WANG | Studies where both the experimental group  and control group received auricular therapy |

Table S3 calculation of parameters for Harbord test

| **Study ID** | **Efficient score (*Z)*** | **Variance of efficient score(*V*)** | **Independent variable (****)** | **Dependent variable ()** |
| --- | --- | --- | --- | --- |
| Baoli ZHANG 2016 | 3.91 | 2.07 | 1.44 | 2.72 |
| Hong HUANG 2025 | 4.00 | 1.72 | 1.31 | 3.05 |
| Hongrui ZHANG 2024 | -0.07 | 0.95 | 0.97 | -0.07 |
| Yanhui ZHANG 2023 | 2.00 | 0.96 | 0.98 | 2.04 |

**Footnotes:**$Z=d_{1}-\frac{d\times n_{1}}{n}$, $V=\frac{n_{0}\times n_{1}\times d\times h}{n^{2}\times\left( n-1 \right)}$, where *d* represented total number of effective cases, $d_{1}$ represented the number of effective cases in the treatment group, *n* represented total number of cases in the treatment and control group, $n_{0}$represented the total number of cases in the control group; $n_{1}$ represented the total number of cases in the treatment group cases, and $h$ represented the total number of ineffective cases in both treatment and control groups(viz., $h$=$n$-*d* ).

Table S4 Result of ANOVA using SPSS

|  | Sum of Squares | *df* | Mean Square | *F* | *P* |
| --- | --- | --- | --- | --- | --- |
| Regression | 3.350 | 1 | 3.350 | 2.650 | 0.245^b^ |
| Residual | 2.528 | 2 | 1.244 |  |  |
| Total | 5.877 | 3 |  |  |  |

Footnotes: a. Dependent Variable: Dependent variable; b. It indicates invalid linear regression model.

Table S5 Coefficients of ANOVA using SPSS

|  | **Unstandardized Coefficients** | | **Standardized Coefficients** | ***t*** | ***P*** |
| --- | --- | --- | --- | --- | --- |
|  | ***B*** | **Std. Error** | ***Beta*** |  |  |
| Constant^#^ | -3.319 | 3.275 |  | -1.014 | 0.417 |
| Independent variable | 4.469 | 2.745 | 0.755 | 1.628 | 0.245 |

Footnotes: #, Since the sample was too small, no obvious small-study effect was detected by Harbord test, which could not be valid evidence to support potential publication bias in this meta-analysis.

Figures


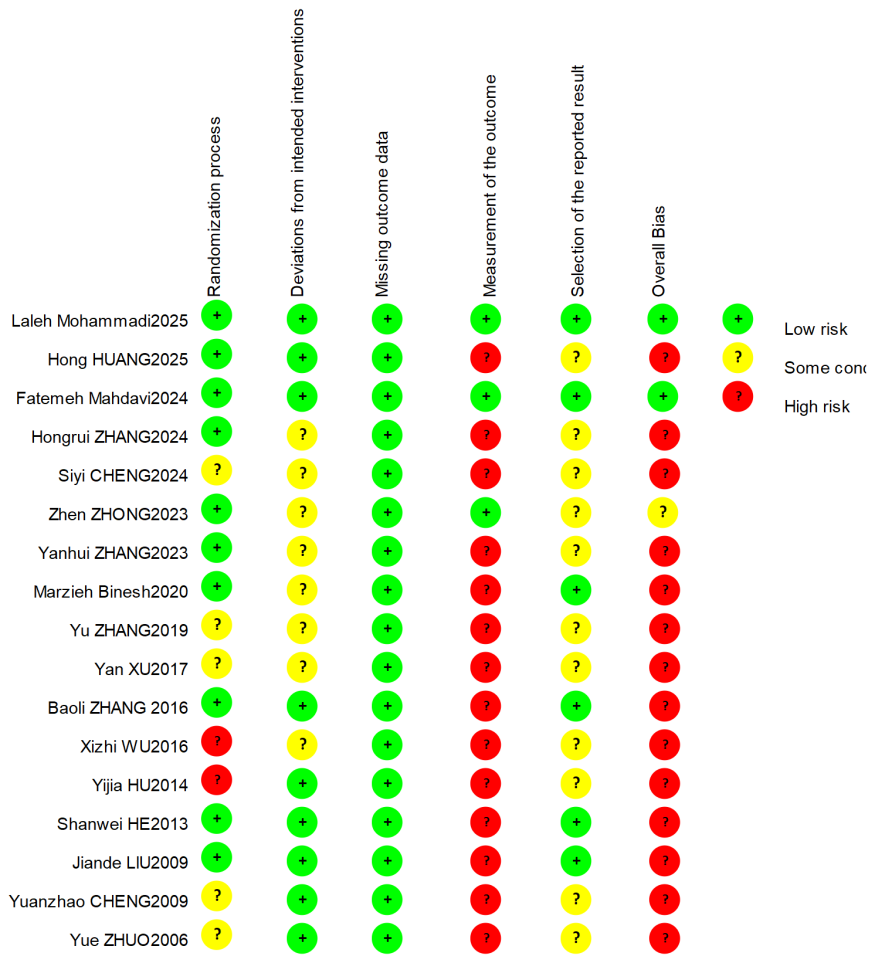


Figure S1 Risk of Bias of included 17 RCTs (detailed graph)


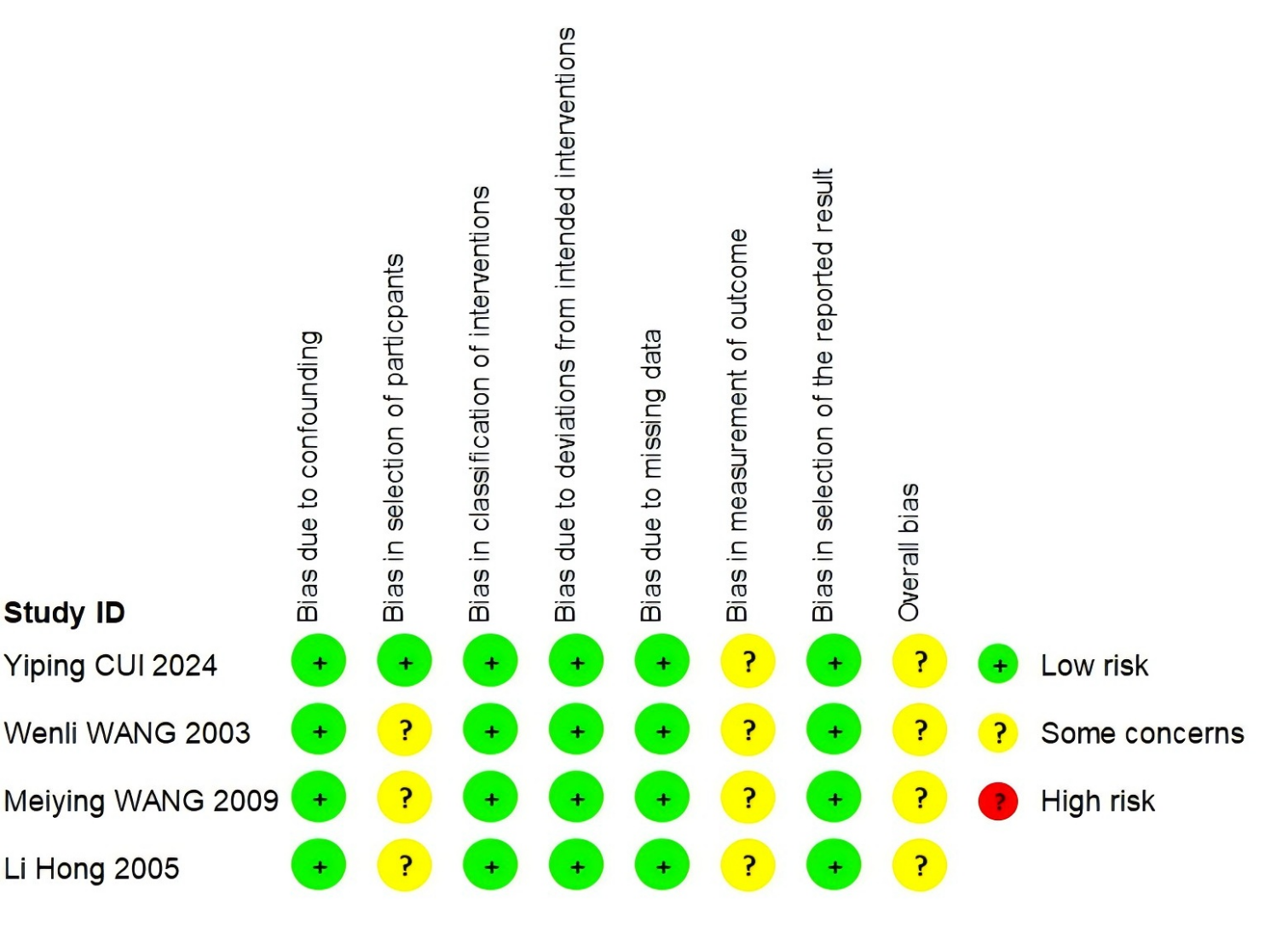


Figure S2 Risk of Bias of included 4 NRCTs (detailed graph)


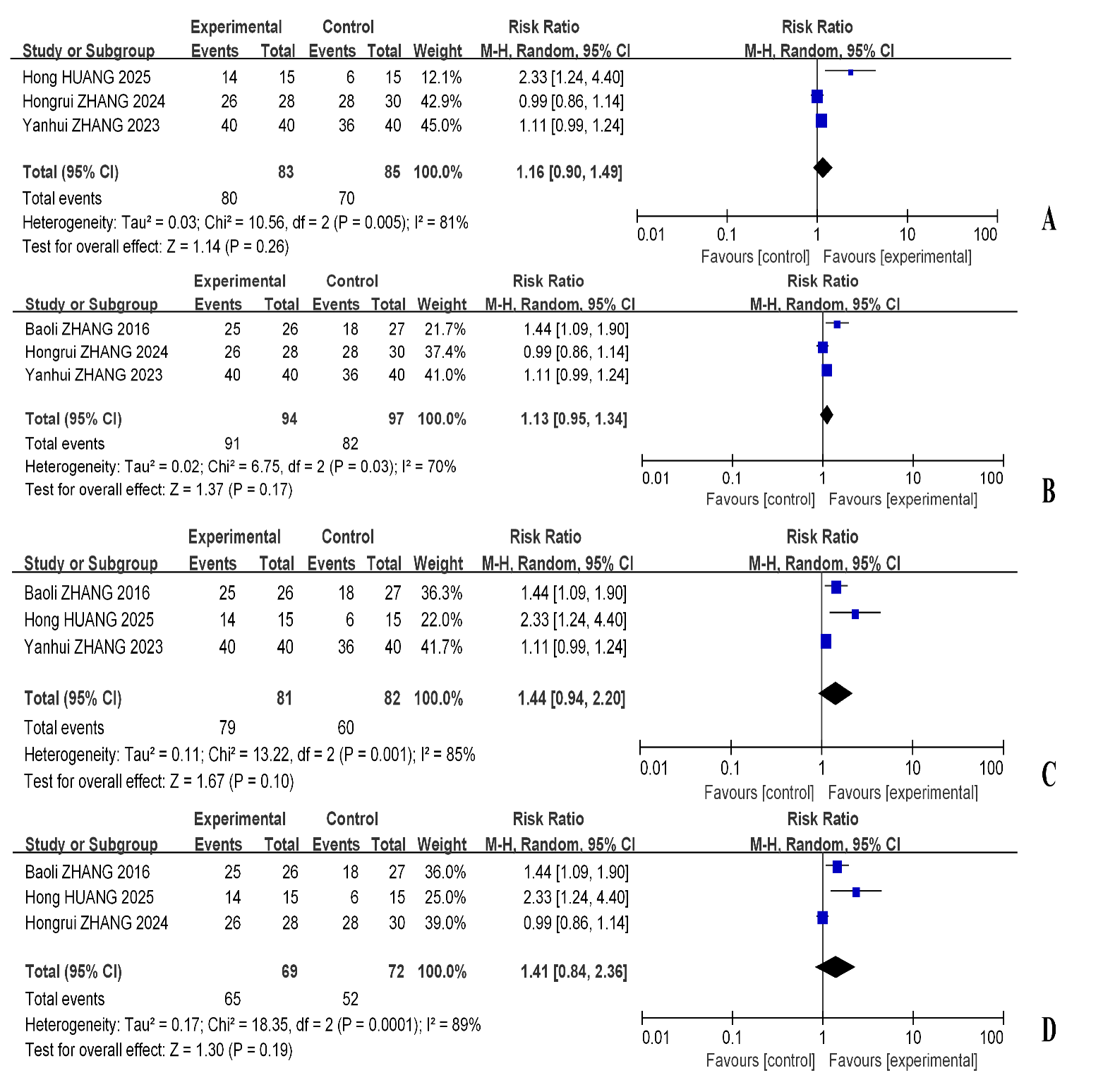


Figure S3 Leave-one-out method for meta sensitivity analysis: auricular therapy + CMs vs. CMs

**A: Meta analysis excepting for Baoli ZHANG; B: Meta analysis excepting for Hong HUANG; C: Meta analysis excepting for Hongrui ZHANG; D: Meta analysis excepting for Yanhui ZHANG;**

**
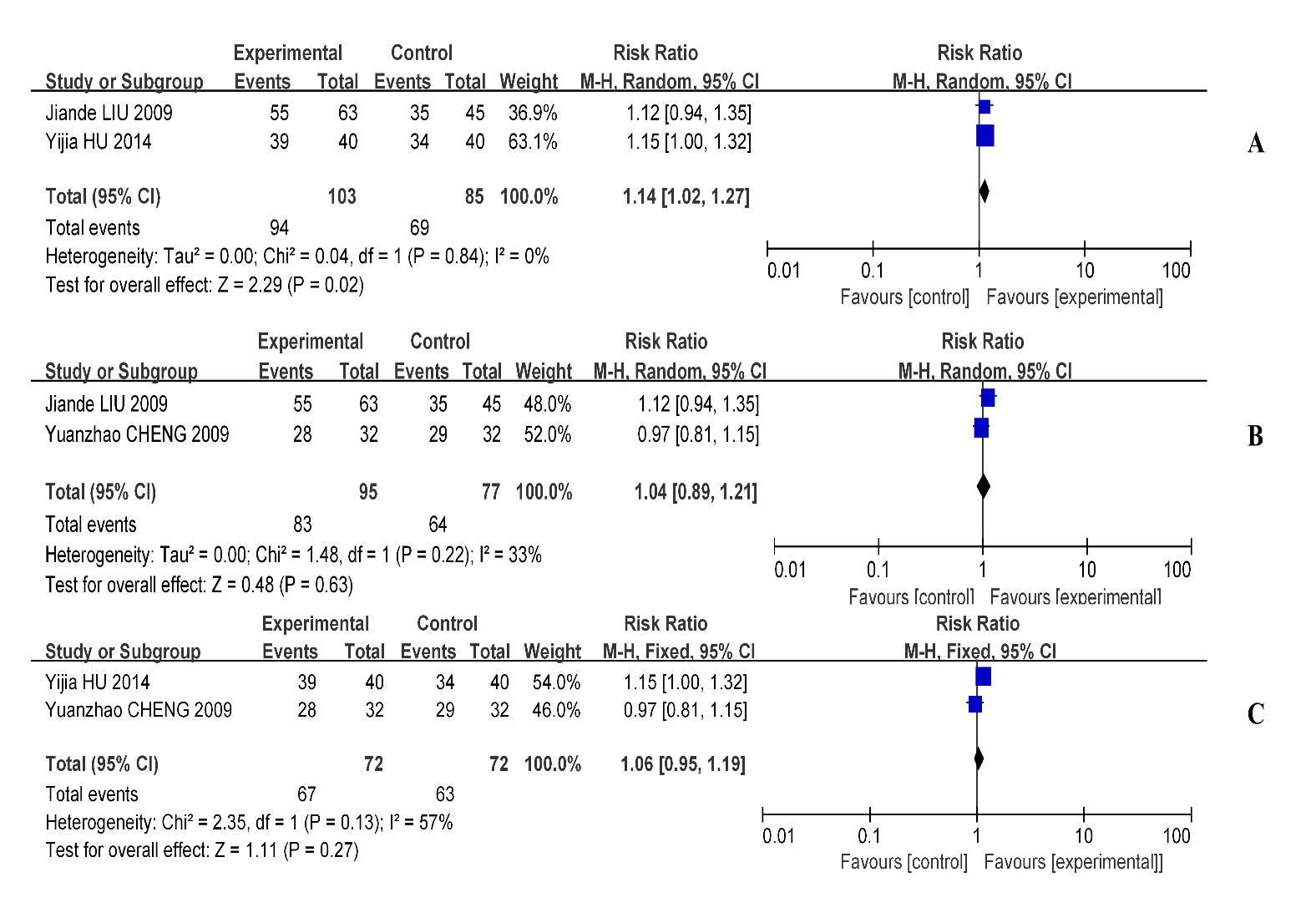
**

Figure S4 Leave-one-out method for meta sensitivity analysis: acupuncture +auricular therapy vs. synthetic drugs
A: Meta analysis excepting for Yuanzhao CHENG; B: Meta analysis excepting for Yijia HU; C: Meta analysis excepting for Jiande LIU

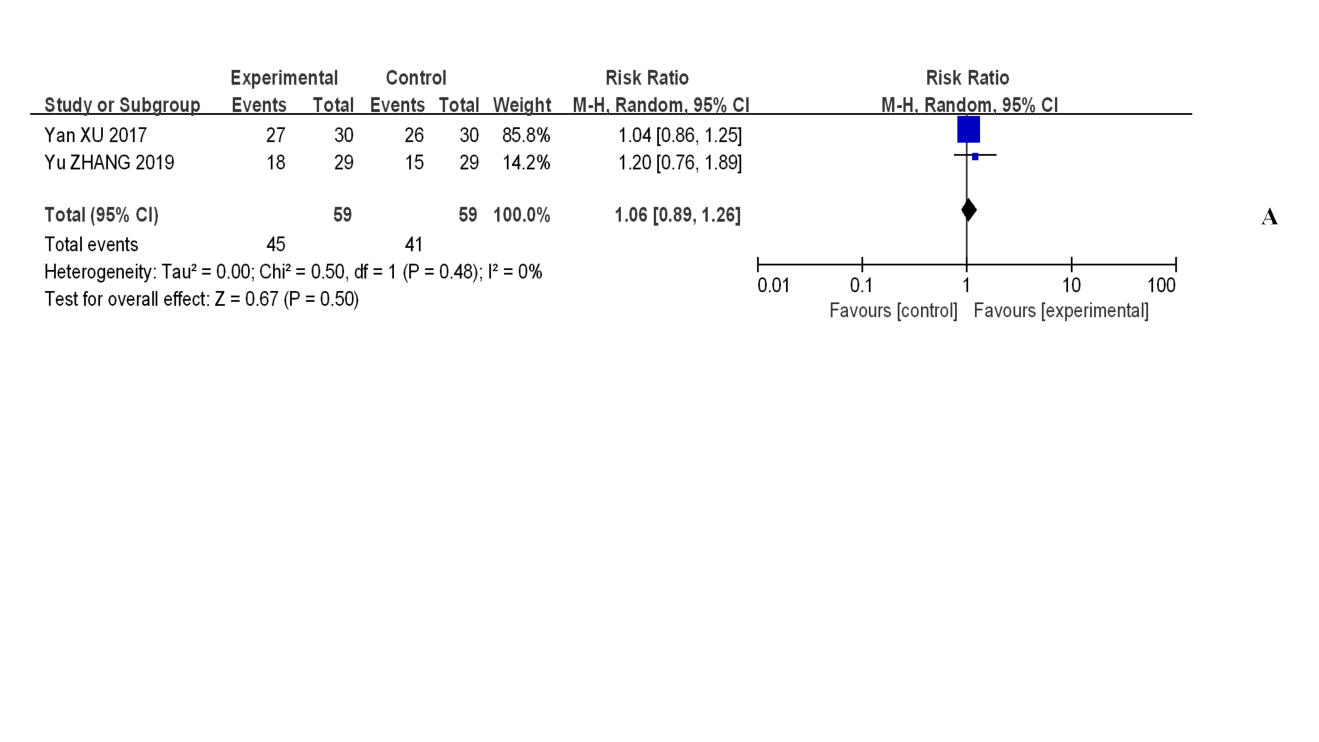
Figure S5 Forest plot of overall efficacy rate: CMs +auricular therapy vs. synthetic drugs

**
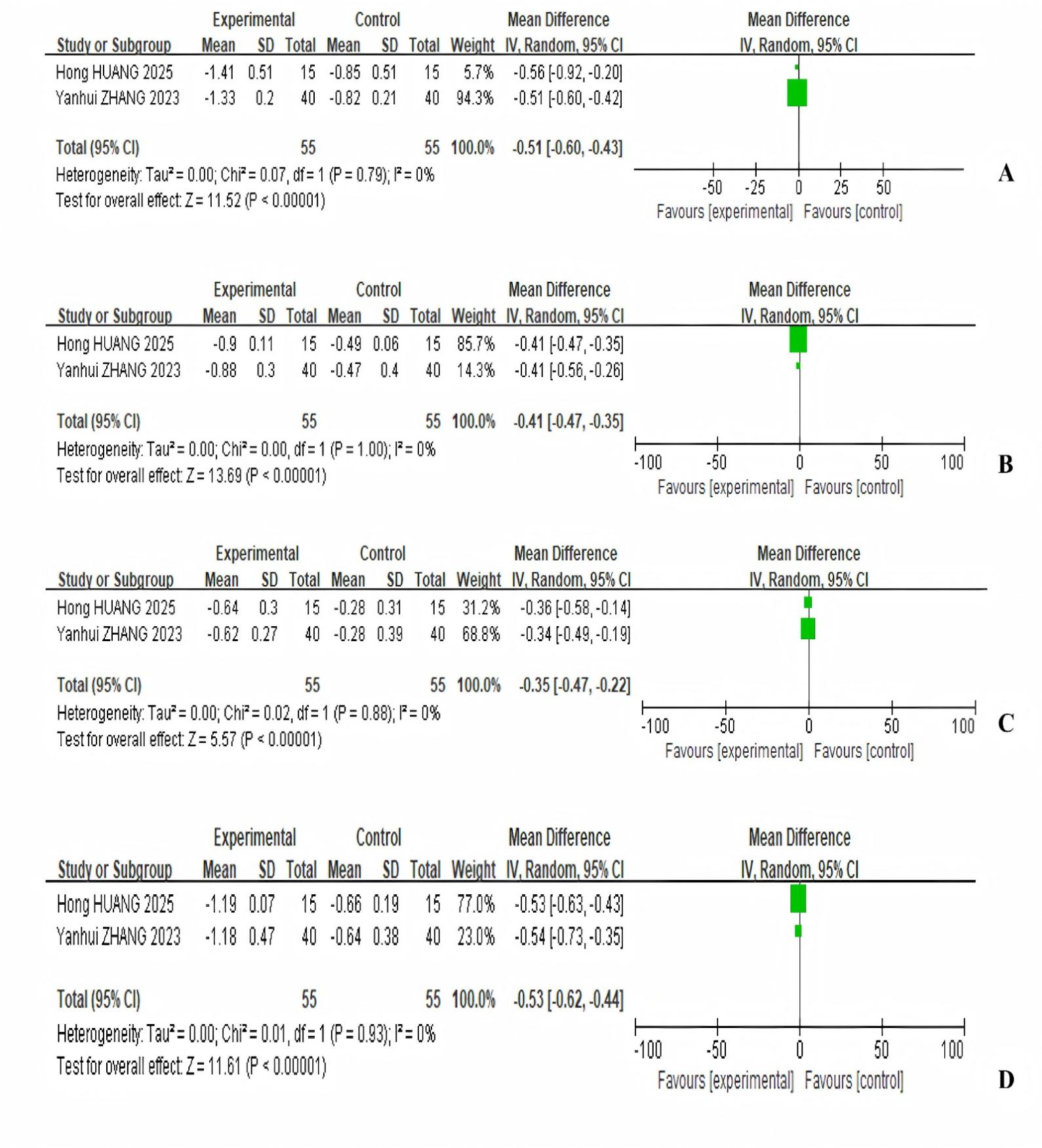
**


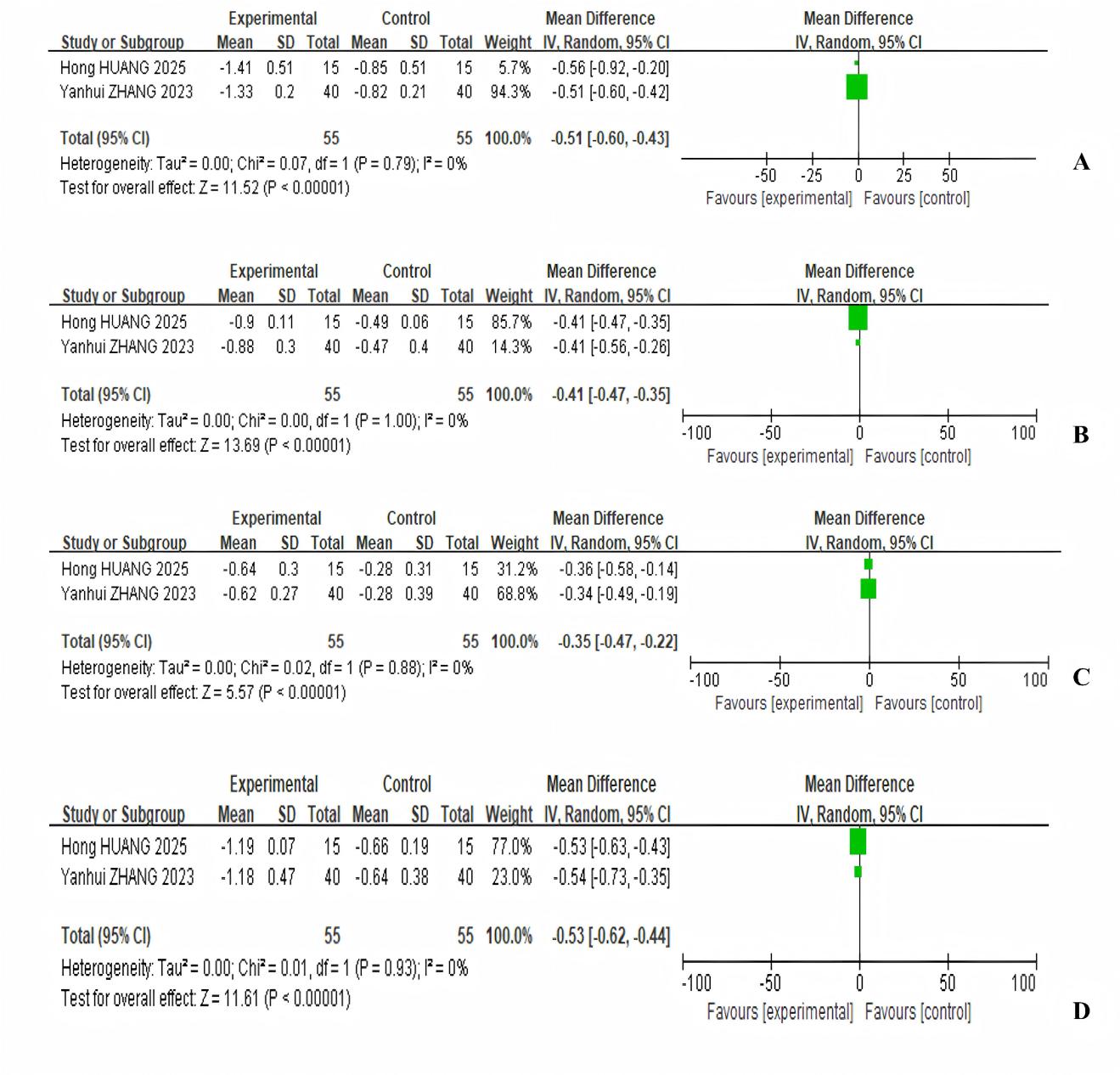
Figure S6 Forest plot of CPRS score: CMs + auricular therapy vs. CMs

**A: Learning problems; B: Psychosomatic problems; C: Conduct problems; D: Impulsivity-hyperactivity.**


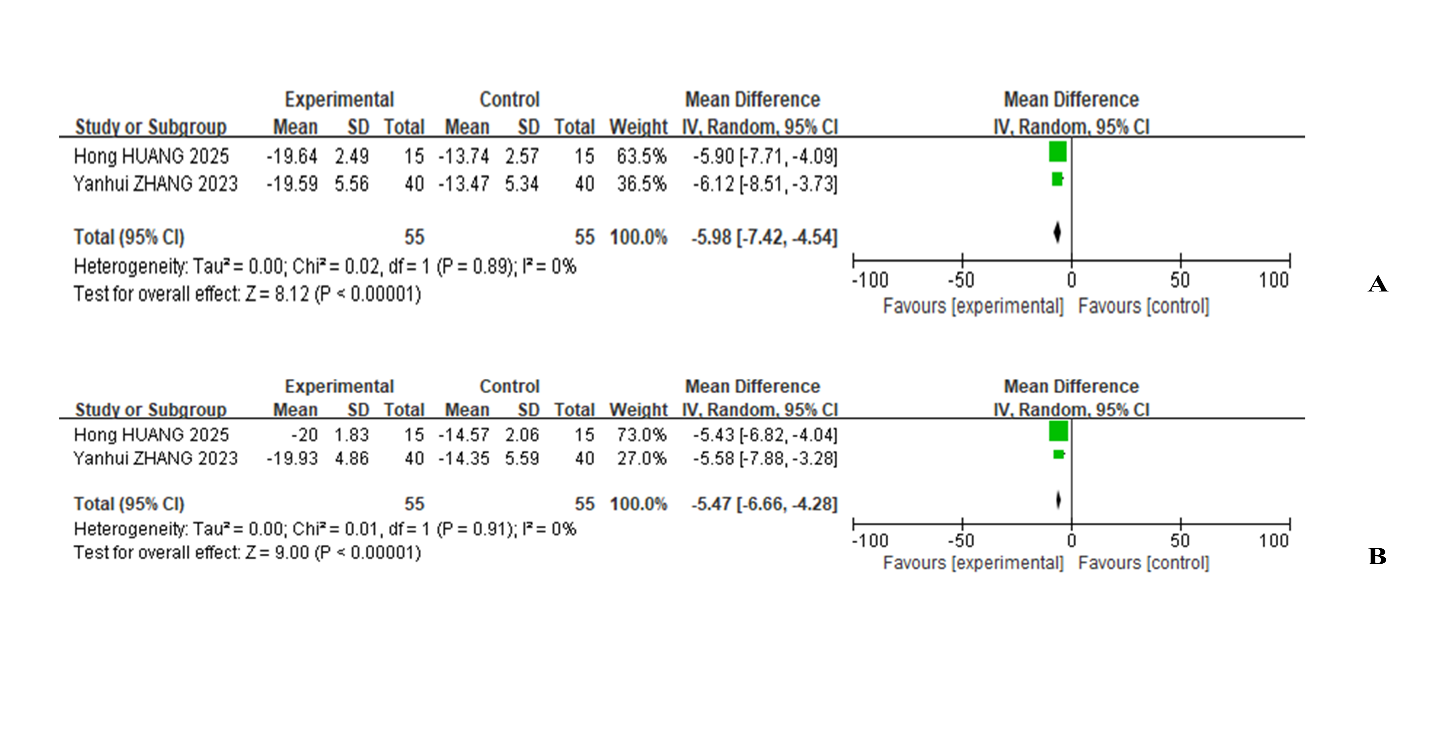


Figure S7 Forest plot of HAMA and HAMD scores: CMs + auricular therapy vs. CMs

**A: HAMA score; B: HAMD score.**


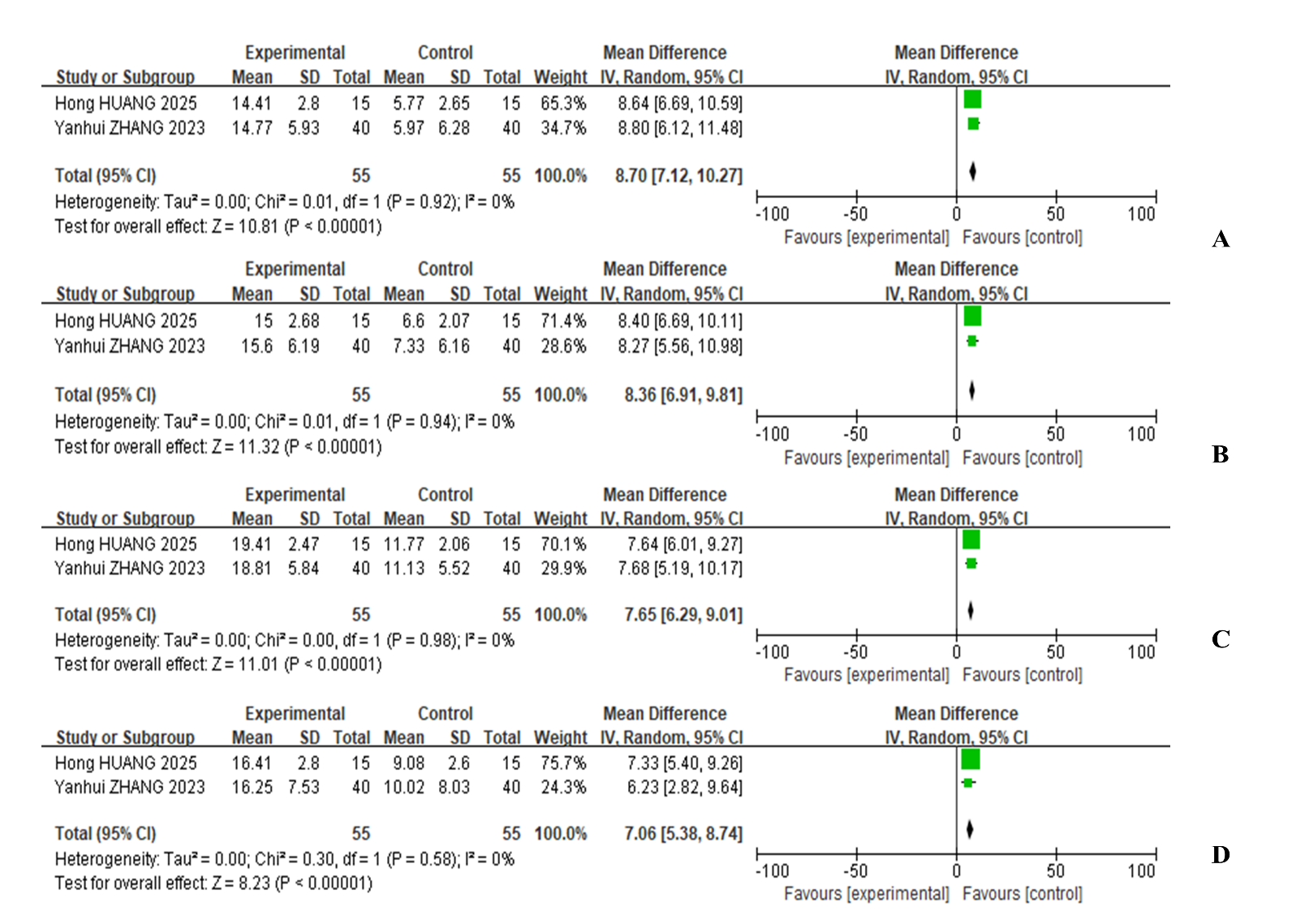


Figure S8 Forest plot of QoL questionnaire score

**A: Physical functioning; B: Social functioning; C: Role-physical domain; D: Mental health.**
